# Geospatial variations in trends of reproductive, maternal, newborn and child health and nutrition indicators at block level in Bihar, India, during scale-up of *Ananya* program interventions

**DOI:** 10.7189/jogh.10.021004

**Published:** 2020-12

**Authors:** Safa Abdalla, Emma Pair, Kala M Mehta1,, Victoria C Ward, Gary L Darmstadt

**Affiliations:** 1 Department of Pediatrics, Stanford University School of Medicine, Stanford, California, USA; 2 Department of Epidemiology and Biostatistics, University of California San Francisco, San Francisco, California, USA; 3 Center for Population Health Sciences, Stanford University School of Medicine, Palo Alto, CA, USA

## Abstract

**Background:**

Geographical variations in the levels and trajectory of health indicators at local level can inform the adaptation of interventions and development of targeted approaches for efficient scale-up of intervention impact. We examined the hypothesis that time trends of a set of reproductive, maternal, newborn, and child health and nutrition (RMNCHN) indicators varied at block-level during the statewide scale-up phase of the *Ananya* program in Bihar, India.

**Methods:**

We used data on 22 selected indicators from four rounds of the Community-based Household Survey carried out between 2014 and 2017. Indicator levels at each round were estimated for each block. We used hierarchical Bayesian spatiotemporal modelling to smooth the raw estimates for each block with the estimates from its neighbouring blocks, and to examine space-time interaction models for evidence of variations in trends of indicators across blocks. We expressed the uncertainty around the smoothed levels and the trends with 95% credible intervals.

**Results:**

There was evidence of variations in trends at block level in all but three indicators: facility delivery, public facility delivery, and age-appropriate initiation of complementary feeding. Fifteen indicators showed trends in opposite directions (increases in some blocks and declines in others). All blocks had at least 97.5% probability of a rise in immediate breastfeeding, early pregnancy registration, and having at least four antenatal care visits. All blocks had at least 97.5% probability of a decline in seeking care for pregnancy complications.

**Conclusions:**

The findings underscore the value of monitoring and evaluation at local level for targeted implementation of RMNCHN interventions. There is a need for identifying systematic factors leading to universal trends, or variable contextual or implementation factors leading to variable trends, in order to optimise primary health care program impact.

**Study registration:**

ClinicalTrials.gov number NCT02726230.

Examining geographical variation in health indicators is highly relevant for programs implementing interventions at scale. It is critical for tracking program performance and prioritising areas where trends are not favourable for intensified efforts. Geospatial analysis could also be used to uncover the presence of program-related factors or contextual factors that differentially act or prevail at the local level to influence the direction of trends, potentially leading to inequitable impact of interventions. Factors which vary by space or geography have included, for example, geographical distribution of services [1], beneficiaries’ geographical access to services [2,3], or safety and mobility factors affecting geographical access of outreach service to beneficiaries [4].

The *Ananya* program is a partnership of the Bill & Melinda Gates Foundation (BMFG) with the Government of Bihar (GoB) in India that aimed to accelerate the achievement of Bihar’s ambitious reproductive, maternal, newborn and child health and nutrition (RMNCHN) goals. The program and its evaluation are described in detail elsewhere [5-10]. Briefly, the program initially piloted a range of select household, community and facility-level interventions, with intensive ancillary support by non-governmental organisations (NGOs) to governmental implementation in eight “focus” districts from 2011 through 2013. The second phase, from 2014 to present, involved a shift from intensive NGO support to the provision of techno-managerial support through the Bihar Technical Support Program (BTSP), emphasising and facilitating government ownership and their own capacity and actors to sustain the interventions and functions introduced and piloted in the initial phase.

Geographical variations in RMNCHN have been reported in India, including Bihar, at district level [11]. District-level changes were also previously examined for a limited set of indicators as part of the *Ananya* program monitoring efforts [12,13]. Apart from an examination of links between frontline worker (FW) functions and complementary feeding indicators in the first phase of the program [14], little is known about variations across RMNCHN at a more local subdistrict/block level. The block is a key administrative level where critical service delivery and implementation management functions take place, and where variations can readily be masked by district averages. More importantly, the trajectories of those indicators and how they vary between blocks during the scale-up phase is unknown. Uncovering such geographical variations at a more local level, at a time when governmental systems should be adapting interventions and adjusting delivery approaches as needed [15], is therefore important. However, it requires careful analysis that accounts for instability of estimates where denominators are small, as well as for various potential sources of errors in raw estimates such as sampling error [16]. We hypothesised that there are geospatial variations of RMNCHN indicators and aimed in this paper to investigate levels and trends of selected RMNCHN indicators at block-level in the second phase of the *Ananya* program using geospatial analysis methods designed for robust small-area estimation. We compare a model that includes space-time interaction (assuming geospatial differences in trends) with one that does not in order to determine the explanatory role of the interaction for the levels of indicators across years and blocks.

## METHODS

### Data source

We used data from the Community-based Household Surveys (CHS), a series of cross-sectional surveys designed and conducted in Bihar by CARE India, from which block-level data were available. We used rounds 6-9 of the survey covering all 38 districts in Bihar from 2014-2017; prior survey rounds were comprised of more limited data from eight focus districts during the pilot phase of the *Ananya* program which focused on testing innovations. Rounds 6-9 were carried out during the second phase when program implementation had been scaled-up to all 38 districts. In this phase, a modified Lot Quality Assurance Sampling-like (LQAS+) methodology was used with a larger sample size aimed to obtain point estimates for each district with a precision of ±5%. The Anganwadi Centre (AWC) was the chosen unit for collating a random sample for each block because the catchment areas of the centres had limited variation in size, and a reliable sampling frame of AWCs was available. After the random selection of AWCs with their catchment areas, eligible participants were identified by systematically identifying households to visit. Starting from one randomly selected household from a list of households kept by the AWC, each 5th household in a clockwise direction was visited until one eligible mother from each child age group was identified and enrolled in the survey. In this way, a random sample of mothers of children younger than age two years was selected from each of 534 blocks, proportionate to the number of eligible mothers in each block [17]. The sampling was stratified by the children’s age group (0-2, 3-5, 6-8, 9-11, and 12-23 months), with a minimum of 19 mothers per age group, yielding approximately 15 687 women per children’s age group per round. To minimise the recall period, the mothers of children in each age group responded to questions in different continuum of care domains that were the focus of interventions for that age group. The sample size at block level ranged from 19 to 123 mothers per age group per survey round.

An interview questionnaire was used to collect data on sociodemographic characteristics and included specific modules for RMNCHN indicators relevant to each age group. Respondents completed an informed consent prior to the interview. Quality control included mandated spot checks and back-checks of 15% of the data by independent field supervisors. A meta-database digital quality control system capable of tracking individual data collection devices in real-time was established in 2016. Fieldwork in each round took about 2-3 months. Rounds 6-9 were conducted with 12-16 months in between rounds, and the data were collected by staff of Care India’s Concurrent Measuring and Learning unit which was functionally independent of implementation.

### Indicators

We assessed antenatal care (ANC), birth preparedness, delivery (childbirth), postnatal and breastfeeding indicators for children aged 0-2 months; and indicators of complementary feeding and use of contraception by mothers of children aged 9-11 months. We selected 22 indicators from across the continuum of RMNCHN. In addition, we classified indicators within each continuum of care domain by delivery platforms: FLW performance, mother’s behaviour, and facility care or outreach service delivery (Table S1 in the **Online Supplementary Document**). Table S2 in the **Online Supplementary Document** describes the selected indicators. All the indicators were binary (yes/no) variables in the individual-level data, except for the number of FLW advice items, number of FLW advice items on birth preparedness, and number of birth preparedness measures taken.

### Analysis

We first estimated raw proportions from numerator and denominator data for each binary yes/no indicator at block level using SAS 9.4. For count indicators, the counts from the individual-level data were averaged over each block, weighted by the maximum possible count, which varied across respondents due to different requirements depending on planned place of birth in the case of birth preparedness measures or the different number of FLW advice items mothers were asked about in round 9 vs the other survey rounds.

To account for unstable estimates due to small numbers as well as sampling and non-sampling errors, we smoothed the estimates and investigated spatiotemporal patterns using hierarchical Bayesian modelling with the Integrated Nested Laplace Approximation method in R (R-INLA: www.r-inla.org) [18,19]. We applied the Besag-York-Mollié (BYM) spatiotemporal model which accounts for spatial correlation in the data where observations in neighbouring areas are likely more similar than observations in areas farther away [20]. The model has a spatial random effect that uses a neighbourhood structure to smooth raw estimates, and an unstructured component that models uncorrelated noise [21,22]. We defined the neighbourhood structure for each block as the blocks that share a border with it. We added a fixed effect for time (representing the state average) and looked for evidence of variations in trend by comparing a model that included a space-time interaction term with another that did not include an interaction term, assuming linear trends in each block. The model with the lower deviance information criterion (DIC) was selected. If the interaction model had the lower DIC, we considered that to be evidence of variation in trends and estimated the trends for each block by adding the fixed time effect and the interaction term. We mapped the trend estimates along with round 6 and round 9 smoothed estimates (presented in Figure S1 in the **Online Supplementary Document**). Binomial models with a logit link were used with yes/no binary indicators, with the trend expressed as relative change in odds ratio (OR) per year. Poisson models with a log link were used with count indicators, expressing the trend as relative change in count per year. Credible intervals (CrI) for the proportions were represented by the 2.5^th^ and 97.5^th^ quantiles of the posterior distribution of the predicted proportions from the smoothing model. The 95% CrIs for the block-level trends were estimated by resampling 10 000 times from the posterior distribution of each of the fixed time effect and the space-time interaction effect, adding the two sampled values in each time. The 2.5^th^ quantile and the 97.5^th^ quantile of the distribution of the 10 000 values obtained represented the block-level trend’s lower and upper credible limits, respectively. A 95% credible interval with all values below 1.0 indicated that there was a 97.5% probability or more that the relative change per year was lower than 1.0, and one with all values falling above 1.0 indicated that there was a 97.5% probability or more that the relative change per year was higher than 1.0. Blocks with missing data (no respondents) for an indicator were included and the selected model simultaneously imputed their estimates.

### Ethical considerations

Permission for access and terms of CHS data use were agreed through a data sharing agreement with CARE India and approved by the Stanford University Institutional Review Board (protocol number 39719). This study is part of the *Ananya* program which was registered with ClinicalTrials.gov number NCT02726230.

## RESULTS

### Indicator levels in survey round 6

**Figure 1:**
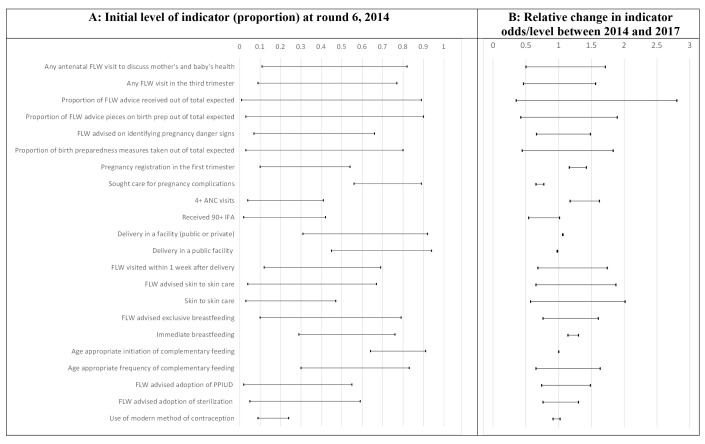
Range of initial levels and trends for each indicator across blocks, Community-based Household Surveys rounds 6-9, 2014-2017 Bihar, India. 95% credible intervals for the minimum and maximum values are reported in Table S3 in the **Online Supplementary Document**. ANC – antenatal care, FLW – frontline worker, IFA – iron and folic acid, PPIUD – post-partum intrauterine device.

**Figure 1** displays the range of smoothed block-level proportions for each indicator in survey round 6 when statewide scale-up had begun across blocks. There was wide block-level variation in the levels of some indicators at the beginning of phase 2 (round 6) of *Ananya*. The smallest variation was in use of modern contraception, which was uniformly low, ranging between 9% (95% credible interval (CrI) = 5%-14%) and 24% (95% CrI = 18%-30%). All other count indicators showed wide variations; for example, the average number of birth preparedness measures as a proportion of the total measures recommended ranged from 3% (95% CrI = 2%-4%) to 80% (95% CrI = 71%-90%) and the average proportion of FLW advice items out of the total promoted by the program ranged from 3% (95% CrI = 2%-5%) to 90% (95% CrI = 76%-100%). The widest variations among the binary indicators were in levels of indicators related to FLW performance, including FLW home visits and number of FLW advice items. Any FLW home visit in the third trimester of pregnancy ranged from 9% to 77% and FLW advice on exclusive breastfeeding ranged between 10% (95% CrI = 4%-18%) and 79% (95% CrI = 69%-88%). The distribution of the levels of most indicators across blocks was either symmetrical or skewed towards the lower end of the levels spectrum except for facility delivery, delivery in a public facility and age-appropriate initiation of complementary feeding which started off in 2014 (round 6) with most blocks having high levels of those indicators in that round (Figure S2 in the **Online Supplementary Document**).

### Overview of trends in indicators across rounds 6-9

**Figure 2:**
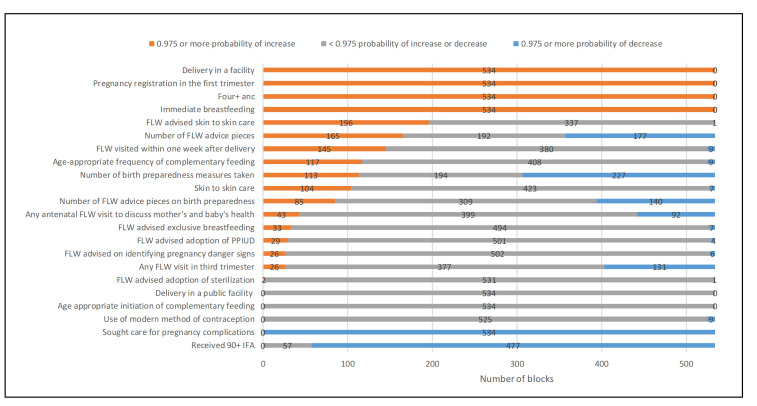
Number of blocks with 97.5% or more probability of an increase in the level of the indicator between 2014 and 2017, Community-based Household Surveys rounds 6-9, Bihar, India. ANC – antenatal care, FLW – frontline worker, IFA – iron and folic acid, PPIUD – post-partum intrauterine device.

**Figure 3:**
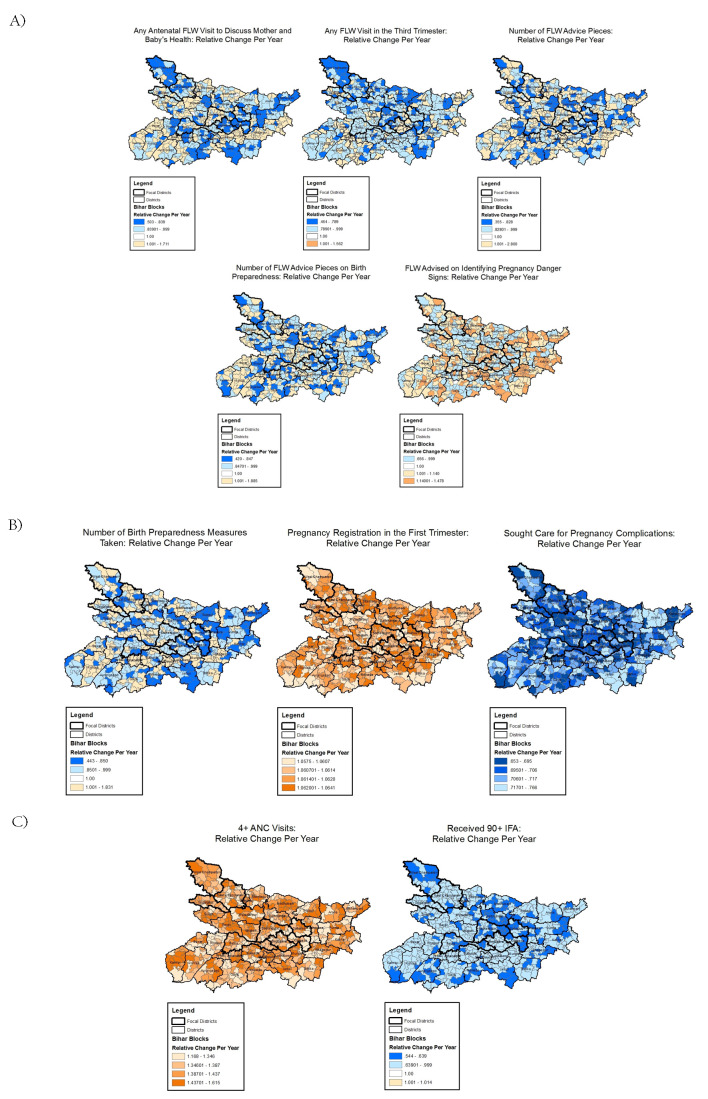
Block-level trends in antenatal care indicators with evidence of space-time interactions, Community-based Household Surveys rounds 6-9, 2014-2017, Bihar, India. **Panel A**. Frontline worker (FLW) performance indicators. **Panel B**. Mother’s behaviour indicators. **Panel C**. Facility / Outreach service delivery indicators. ANC – antenatal care, IFA – iron and folic acid.

**Figure 4:**
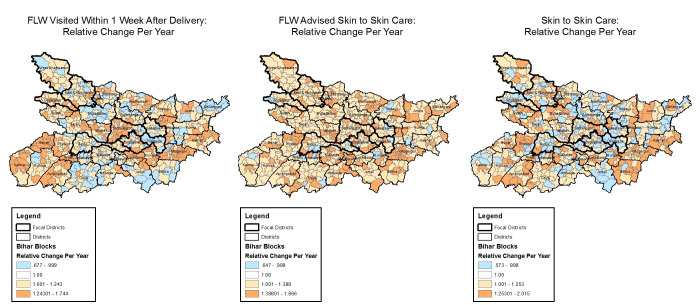
Block-level trends in postnatal care indicators with space-time interactions, Community-based Household Surveys rounds 6-9, 2014-2017, Bihar, India. FLW – frontline worker.

**Figure 5:**
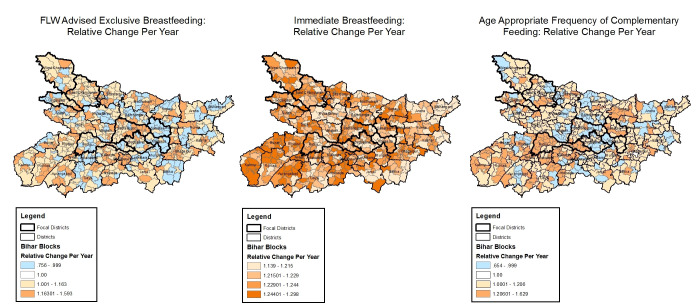
Block-level trends in nutrition indicators with space-time interactions, Community-based Household Surveys rounds 6-9, 2014-2017, Bihar, India. FLW – frontline worker.

**Figure 6:**
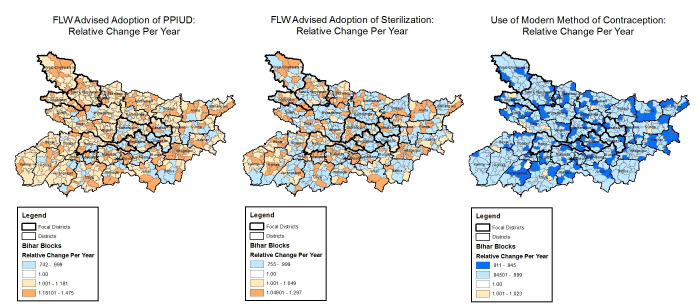
Block-level trends in family planning indicators with space-time interactions, Community-based Household Surveys rounds 6-9, 2014-2017, Bihar, India. FLW – frontline worker, PPIUD – post-partum intrauterine device.

**Figure 1** displays the range of block-level trend estimates. Among all indicators examined, only three did not show evidence of variation in trends between blocks (ie, the model with a space-time interaction was not superior to the one with no interaction): facility delivery, delivery in a public facility and age-appropriate initiation of complementary feeding. **Figure 2** shows the number of blocks with a 97.5% or more probability of an increasing or a declining trend. All blocks had a 97.5% or more probability of a rise in odds with respect to four indicators: delivery in a facility, early pregnancy registration, having four or more ANC visits, and immediate breastfeeding. None of the blocks had a 97.5% or more probability of a rise in receiving 90+ IFA tablets, use of a modern method of contraception, delivering in a public facility, age-appropriate initiation of complementary feeding, or seeking care for complications. All blocks had a 97.5% or more probability of a decrease in the odds of the latter. An additional summary of the findings is also given in Figure S3 in the **Online Supplementary Document**, mapping the number of indicators where the relative change was more than 1.0 or less than 1.0 with 97.5% or more probability. Figures 3-6 display maps of block-level trends for the indicators with evidence of variation in trends by block; note that dark borders enclose the eight districts that were the focus of the pilot phase.

### Variations in trends across continuum of care domains

#### Antenatal care

Receiving at least one FLW health-focused, antenatal home visit in the third trimester showed variable trends, with a decline in the odds by more than 50% per year in one block (OR = 0.46, 95% CrI = 0.36-0.59) and an increase in odds by more than 50% per year in another block (1.56, 95% CrI = 1.23-1.97). Similarly, the total number of advice items received by the mother from a FLW showed opposite trends in different blocks: the maximum decline in count was 65% per year (OR = 0.35, 95% CrI = 0.30-0.41) and the maximum rise was 180% per year (OR = 2.80, 95% CrI = 2.21-3.55). Concerning birth preparedness promotion, the number of advice items also declined by as much as 58% (OR = 0.42, 95% CrI = 0.33-0.52) per year in one block and increased by as much as 88% (OR = 1.88, 95% CrI = 1.57-2.27) in another (**Figure 3**, Panel A). Similarly, wide variation was evident for receiving FLW advice on at least one pregnancy danger sign.

Among trends in mothers’ behaviours in the antenatal period, trends in pregnancy registration in the first trimester and seeking care for pregnancy complications varied among blocks but all blocks experienced trends in the same direction – increasing for the former indicator and decreasing for the latter (**Figure 1**, Panel B). In contrast, the average number of birth preparedness measures taken showed block-level trends in opposite directions; the largest decline in odds was 56% per year (OR = 0.44, 95% CrI = 0.38-0.51) and the largest increase was 83% per year (OR = 1.83, 95% CrI = 1.68-1.99) (**Figure 3**, Panel B).

Trends in antenatal facility/outreach service delivery indicators were mostly in the same direction although varying in magnitude; having at least four ANC visits increased in all blocks with odds ranging from 1.17 (95% CrI = 1.02-1.32) to 1.62 (95% CrI = 1.42-1.84). In all blocks, receiving 90+ IFA decreased or did not change, with odds ranging from 0.54 (95% CrI = 0.41-0.70) to 1.01 (95% CrI = 0.83-1.23) (**Figure 3**, Panel C).

#### Postnatal care

Indicators of postnatal care showed trends that were highly variable across blocks, taking different directions in some blocks and remaining stationary in other blocks (Figure 4). Block-level trends in odds for a FLW visit in the first week ranged from 0.68 (95% CrI = 0.53-0.85) to 1.74 (95% CrI = 1.38-2.21), for FLW advising skin-to-skin care from 0.65 (95% CrI = 0.51-0.81) to 1.87 (95% CrI = 1.49-2.36), and for the practice of skin-to-skin care from 0.57 (95% CrI = 0.45-0.72) to 2.02 (95% CrI = 1.56-2.65).

#### Nutrition

Among indicators of nutrition, FLW advice on exclusive breastfeeding and age-appropriate frequency of complementary feeding had block-level trends that varied in opposite directions (**Figure 5**), with ORs for the former indicator ranging from 0.76 (95% CrI = 0.59-0.96) to 1.59 (95% CrI = 1.22-2.10) and for the latter indicator ranging from 0.65 (95% CrI = 0.53-0.80) to 1.63 (95% CrI = 1.32-1.20). In contrast, the level of immediate breastfeeding increased in all blocks, with variations in the magnitude of the increase: OR range 1.14 (95% CrI = 1.04-1.24) to 1.30 (95% CrI = 1.19-1.44)].

#### Family planning

The yearly change in odds of FLW advice on the adoption of sterilisation varied across blocks from 0.76 (95% CrI = 0.62-0.90) to 1.30 (95% CrI = 1.08-1.57) and the yearly change in odds of FLW advice on the use of a postpartum intrauterine device ranged from 0.74 (95% CrI = 0.57-0.95) to 1.47 (95% CrI = 1.18-1.84). On the other hand, use of a modern method of contraception by mothers showed no evidence of an increase in any of the blocks (**Figure 6**).

## DISCUSSION

We examined block-level variations in levels and trends of a range of 22 RMNCHN indicators, finding evidence of variations in trends at block level in 19 of those indicators from across the continuum of care. There was evidence of opposite trends in different blocks in 15 indicators, which also involved blocks within the same district. There was evidence of a universal increase in four indicators and a universal decline in one other indicator.

These results add to our previous work examining statewide trends in a range of RMNCHN indicators among the least and most marginalised [23], and expand on our previous report of trends in statewide averages of RMNCHN indicators over the pilot and scale-up phases of *Ananya* [7]. We show here that average trends masked considerable differences in block-level trends, even within the same district, in a way that may invalidate any assumptions about what could have been driving change based only on the average statewide or even district-level trends. For some indicators, some blocks were not improving as fast as other blocks even when there was an almost universal positive trend across blocks. Those situations are less alarming than when there were almost universal declines in the indicator, or when indicators were improving in some blocks but declining in others. The former situation could be due to systemic factors overshadowing the effects or impeding implementation of the *Ananya* interventions, adoption of practices, or access to services, requiring universal approaches across blocks. The latter situation could be due to local, spatially varying contextual factors or factors related to the implementation of *Ananya* interventions, or a combination of those, acting independently or interacting with each other, requiring locally varying, targeted approaches. With a dearth of studies exploring geospatial variations in RMNCHN indicator trends, we can only hypothesise about the possible drivers of the patterns we found, based on known links between them and the indicators examined here, with the need for future research to test these hypotheses.

It is notable that the indicators for which there was no evidence of trend variation at block level (ie, delivering in a facility, delivery in a public facility and age-appropriate initiation of complementary feeding) were also the ones where most blocks had initial levels in 2014 at the higher end of the spectrum, experiencing minimal change over survey rounds 6-9. This may point to the possibility of long-standing government programs, such as those that promote institutional delivery [24], producing results statewide and leading to most blocks having already achieved high levels by 2014 (ie, survey round 6) with little room for further increases. The fact that these levels did not drop may indicate that these efforts continue to be effective and/or the prevalence of these practices exceeded a tipping point after which they became very common and therefore self-sustaining as community norms [25].

Immediate breastfeeding, pregnancy registration, and having four or more ANC visits increased in all blocks across the state, with some variation across blocks in the extent of this increase. Supporting immediate breastfeeding has been an important aspect of building the capacity of birth attendants in public facilities [26]. With a high level of facility deliveries, including in public facilities, in most blocks, this finding may be linked to and reflect the outcome of those capacity-building efforts. Having four or more ANC visits is one of three indicators (others being receiving 90+ IFA and use of a modern method of contraception) where the highest levels recorded for a block were in fact the lowest compared with other indicators. Four or more ANC visits was also the only indicator among these three indicators to show a universally favourable trend. Therefore, this is a welcome change, but whether it is driven by increasing contact with FLWs early in pregnancy for the purpose of pregnancy registration or other interventions remains to be uncovered.

Seeking care for complications, receiving 90+ IFA and use of a modern method of contraception did not show evidence of an increase in any of the blocks, with the former declining in all blocks. Factors such as financial accessibility, transport availability, disrespectful treatment [27], and perceived poor quality of health facilities in terms of infrastructure, lack of equipment, supplies and/or personnel were previously linked with utilisation of maternal care services [28]. However, it is unclear which of these factors or others could have contributed to lack of improvements in seeking care for complications. The pattern seen with receiving 90+ IFA and modern contraception use may have been due to statewide issues in the supply chain. In particular, the trend in receiving an adequate supply of IFA are contrary to what would be expected given the increase in ANC visits and the link found between that and better IFA receipt and consumption [29]. Major issues had previously been identified with the IFA supply chain in Bihar [30], which may have contributed to these findings. Strong social norms may also have interfered with progress in use of modern methods of contraception [31].

Indicators of FLW performance in the antenatal and postnatal periods displayed opposite trends across blocks. We are unaware of similar studies looking into variations in trends in FLW performance but several studies linked community health workers’ performance with individual factors such as having a supportive spouse or family [32], community factors such as cultural norms, safety and security, educational level of beneficiaries, and health system factors such as health service functioning, human resource provisions, levels of decision-making and costs of health services [33]. Other factors affecting FLW performance have included community health team-based goals and incentives, intensity and style of supervision and training, community involvement and strong co-ordination and communication with health professionals [34-39].

Similar to indicators of FLW performance, some indicators of mothers’ behaviour – including number of birth preparedness measures adopted, skin-to-skin care, and age-appropriate frequency of complementary feeding – had increased levels in some blocks and decreased levels in others. Variations in birth preparedness measures could be influenced by FLW visits and advice on birth preparedness or by variations in contextual socioeconomic and cultural factors that support preparedness [40]. Currently, skin-to-skin care is promoted primarily in health facility deliveries for high-risk, preterm infants [41]. Geographical variations in those categories could partly underlie variations in the practice. Efforts to test skin-to-skin care as a community-based intervention carried out by mothers at home were previously made in Bangladesh and India [42-44], with facility-centric scale-up currently under way in several states in the latter [45], but there were several barriers to its implementation in very low income settings [46,47]. This could have also contributed to variations in levels and trends in the practice. Local socioeconomic conditions were previously shown to be associated with complementary feeding frequency in India and may be playing a role in the patterns seen with this indicator [48].

The strengths of this study include a unique look into longitudinal trends across blocks for a range of RMNCHN indicators across the continuum of care and delivery platforms, and use of a methodology that accounts for instability of estimates due to small survey denominators at block-level. However, we note a few limitations. We did not account for changes in contextual factors such as wealth or education at block-level and therefore cannot rule out their role in these trends, which limits inferences about health system performance from these results; however, further work is currently under way to explore the role of contextual and health system-related drivers of these trends. Another limitation is that we assumed in the analysis that the trend was linear in all blocks. While this may not be the case in the long run, assuming otherwise is difficult due to the relatively short, four-year time period covered by this analysis. In addition, CHS data are based on mother’s reports, and absence of geo-variations and local trends in reporting tendencies or recall cannot be confirmed.

## CONCLUSION

Trends in several RMNCHN indicators varied geospatially in Bihar during the scale-up of *Ananya* interventions. Where trends are declining universally, a common factor or set of factors – contextual and/or programmatic – could be responsible and needs to be identified and addressed in a universal approach. Where trends are moving in opposite directions, specific local factors and variations therein could be responsible. In this instance, program design and implementation that are fine-tuned and adapted to take into account these local variations and target areas in most need of resources, are likely to be more efficient and effective. This underscores the value of monitoring and evaluation at local levels of health systems – potentially at the lowest level amenable to variations in implementation, such as the block in India. Further work should aim to utilise the variability uncovered in this work, for example by comparing the best vs the worst performing blocks, to understand conditions that predict these trends, which can then inform more targeted approaches to improving health systems performance.

## Additional material

Online Supplementary Document
