# Supplementary material for: Geospatial variations in trends of reproductive, maternal, newborn and child health and nutrition indicators at block level in Bihar, India, during scale-up of Ananya program interventions
Source: J Glob Health. 2020 Oct 23;10(2):021004. doi: 10.7189/jogh.10.021004 (PMC7758914; doi:10.7189/jogh.10.021004)

Geospatial variations in reproductive, maternal, newborn and child health and nutrition indicators in Bihar, India, during scale-up of *Ananya* programme interventions

Table S1: Indicator classification

	FLW	Mother	Facility / Outreach service delivery
**Antenatal care**	Any antenatal FLW visit to discuss mother's and baby's health<br>Any FLW visit in the third trimester<br>Number of FLW advice<br>Number of FLW advice on birth preparedness<br>At least one FLW advice on identifying pregnancy danger signs	Number of birth preparedness measures taken<br>Sought care for pregnancy complications<br>Pregnancy registration in the first trimester	4+ ANC visits<br>Received 90+ IFA
**Delivery**	Delivery in a facility (public or private)<br>Delivery in a public facility (if delivered in a facility)		
**Postnatal care**	FLW visited within 1 week after delivery<br>FLW advised skin-to-skin care	Skin to skin care	
**Nutrition**	FLW advised exclusive breastfeeding	Immediate breastfeeding<br>Age appropriate initiation of complementary feeding<br>Age appropriate frequency of complementary feeding	
**Family planning**	FLW advised adoption of postpartum IUD<br>FLW advised adoption of sterilisation	Use of modern method of contraception	

Table S2: Indicator description

Indicator	Child age group (months)	Indicator description
Any antenatal FLW visit to discuss mother's and baby's health	0-2	ASHA/AWW/ANM ever visited the mother to talk to her about her health or her baby's health
Any FLW visit during third tri-mester	0-2	ASHA/AWW/ANM ever visited the mother to talk to her about her health or her baby's health at least once in the last trimester of pregnancy
Number of FLW advice pieces	0-2	Based on the ASHA/AWW/ANM's advice on any of the following: birth preparedness (detailed below), pregnancy danger signs (detailed below),person conducting the delivery should wash their hands, clean or sterilized thread should be used to tie the cord, new blade should be used to cut cord, nothing should be applied to the cord stump at birth, the baby should be wiped dry with a clean cloth immediately after birth, the baby should be placed naked on the mother's bare chest, the baby should be breastfed as soon as possible after delivery, the baby should not be given anything apart from breast milk in the first six months, the baby should not be bathed in the first 2 days after birth, the need to delivery in hospital
Number of FLW advice pieces on birth preparedness	0-2	Based on the ASHA/AWW/ANM's advice on any of the following: identifying hospital to go to in case of emergency, pre-identifying a vehicle to reach the hospital for delivery or in the case of emergency, keeping money aside for delivery or emergencies, identifying someone to accompany her to hospital, identifying someone willing to donate blood in case of emergency, being prepared for the eventuality of a home delivery, and identifying a trained person to be present in case the delivery had to take place at home.
FLW advised on identifying pregnancy danger signs	0-2	ASHA/AWW/ANM told the mother about any of the following: the danger of excessive bleeding during pregnancy, during delivery or after delivery, the danger of convulsions, the danger of prolonged or difficult labor, and the danger of swelling of the face or hands
Number of birth preparedness measures taken	0-2	Based on the following measures: saving money for delivery or emergency, chosen facility for deliver, chosen facility in emergency situation, arranged transportation to facility, arranged for someone to accompany them, identified someone who will take care of the baby immediately after delivery, identified someone who will donate blood in case of emergency, identified and informed anyone who would assist with delivery, and prepared Disposable Delivery Kit, a new blade, clean thread, soap, a clean cloth for the child, and clean pads for her.
Pregnancy registration in the third trimester	0-2	Mother registered her pregnancy no later than the first three months of pregnancy.
Sought care for complications	0-2	Mother consulted anybody or sought treatment if she had experienced excessive bleeding or convulsions
4+ ANC visits	0-2	Had four or more antenatal care visits during last pregnancy
Received 90+ IFA	0-2	In the last pregnancy, mother received at least 90 tablets of Iron (IFA)

Indicator	Child age group (months)	Indicator description
Place of delivery: In a facility (public or private)	0-2	Delivery took place in Medical college, District hospital, SDH, PHC, APHC/subcenter, or private hospital or clinic
Facility delivery took place in a public facility	0-2	Facility delivery took place in Medical college, District hospital, SDH, PHC, or APHC/subcenter
FLW visited within 1 week after delivery	0-2	At least one visit by any FLW (ASHA, ANM, AWW) in the first week of delivery.
FLW advised skin to skin care	0-2	
Skin to skin care	0-2	Mother was advised by anyone at any time during or after delivery to keep the baby naked on her bare chest, next to your skin, and practiced keeping the baby in that position immediately after delivery/during your stay in the hospital (for facility deliveries)
FLW advised exclusive breastfeeding	0-2	ASHA/AWW/ANM told the mother that the baby should not be given anything other than breastmilk up to the age of six months
Immediate breastfeeding	0-2	Breastfeeding initiated within one hour after birth
Age-appropriate initiation of complementary feeding	9-11	First giving cereal-based semisolid food at age 6 – 8 months
Age appropriate frequency of complementary feeding	9-11	Baby given cereal-based semisolid food 3 – 4 times per day
FLW advised adoption of PPIUD post-delivery	0-2	ASHA/AWW/ANM told the mother that she can adopt PPIUD immediately after delivery to delay next pregnancy
FLW advised adoption of sterilization	0-2	ASHA/AWW/ANM told the mother that she can adopt TL (female sterilization) immediately after delivery to avoid any further pregnany
Modern method of contraception used	9-11	Currently using one of the following methods:<br>1. Male sterilization<br>2. Female sterilization (TL)<br>3. IUD/Loop<br>4. Injectables<br>5. Condom/Nirodh<br>6. Contraceptive pills<br>7. Others (specify)

Table S3: Minimum and maximum initial levels and trend estimates at block-level with 95% credible intervals (CrI), 2014-2017 Bihar, India

**Indicator**	**Initial level of indicator (proportion) at round 6 with 95% CrI**		**Relative change in indicator level between 2014 and 2017 with 95% CrI**	
	**Minimum**	**Maximum**	**Minimum**	**Maximum**
Any antenatal FLW visit to discuss mother's and baby's health	0.11 (0.06 – 0.16)	0.82 (0.72 – 0.90)	0.50 (0.39 – 0.64)	1.71 (1.31 – 2.25)
Any FLW visit in the third trimester	0.09 (0.04 – 0.15)	0.77 (0.68 – 0.84)	0.46 (0.36 – 0.59)	1.56 (1.23 – 1.97)
Number of FLW advice pieces received	0.01 (0.006 – 0.014)	0.89 (0.81 – 0.98)	0.35 (0.30 – 0.41)	2.80 (2.21 – 3.55)
Number of FLW advice pieces on birth preparation received	0.03 (0.02 – 0.05)	0.90 (0.76 – 1.05)	0.42 (0.33 – 0.52)	1.89 (1.59 – 2.27)
FLW advised on identifying pregnancy danger signs	0.07 (0.02 – 0.13)	0.61 (0.48 – 0.74)	0.66 (0.50 – 0.84)	1.48 (1.15 – 1.90)
Number of birth preparedness measures taken	0.03 (0.02 – 0.04)	0.80 (0.71 – 0.90)	0.44 (0.38 – 0.51)	1.83 (1.68 – 1.99)
Pregnancy registration in the first trimester	0.10 (0.06 – 0.14)	0.54 (0.47 – 0.61)	1.16 (1.05 – 1.27)	1.42 (1.29 – 1.57)
Sought care for pregnancy complications	0.56 (0.49 – 0.63)	0.89 (0.85 – 0.93)	0.65 (0.59 – 0.72)	0.77 (0.70 – 0.85)
4+ ANC visits	0.04 (0.03 – 0.07)	0.41 (0.33 – 0.49)	1.17 (1.02 – 1.32)	1.62 (1.42 – 1.84)
Received 90+ IFA	0.02 (0.004 – 0.40)	0.42 (0.33 – 0.52)	0.54 (0.41 – 0.70)	1.01 (0.83 – 1.23)
Delivery in a facility (public or private)	0.31 (0.22) – 0.40)	0.92 (0.87 – 0.95)	1.057 (1.024 – 1.084)	1.064 (1.038 – 1.096)
Delivery in a public facility	0.45 (0.37 – 0.54)	0.94 (0.91 – 0.97)	0.97 (0.95 – 1.00)	0.98 (0.95 – 1.01)
FLW visited within 1 week after delivery	0.12 (007 – 0.18)	0.69 (0.61 – 0.77)	0.68 (0.53 – 0.85)	1.74 (1.38 – 2.21)
FLW advised skin to skin care	0.04 (0.01 – 0.08)	0.67 (0.53 – 0.80)	0.65 (0.51 – 0.81)	1.87 (1.49 – 2.36)
Skin to skin care	0.03 (0.01 – 0.06)	0.47 (0.36 – 0.57)	0.57 (0.45 – 0.72)	2.02 (1.56 – 2.65)
FLW advised exclusive breastfeeding	0.10 (0.04 – 0.18)	0.79 (0.69 – 0.88)	0.76 (0.59 – 0.96)	1.59 (1.22 – 2.10)
Immediate breastfeeding	0.29 (0.22 – 0.36)	0.76 (0.68 – 0.83)	1.14 (1.04 – 1.24)	1.30 (1.19 – 1.44)
Age appropriate initiation of complementary feeding	0.64 (0.55 – 0.72)	0.91 (0.86 – 0.94)	1.00 (0.97 – 1.03)	1.01 (0.98 – 1.05)
Age appropriate frequency of complementary feeding	0.30 (0.20 – 0.40)	0.83 (0.73 – 0.90)	0.65 (0.53 – 0.80)	1.63 (1.32 – 2.03)
FLW advised adoption of PPIUD	0.02 (0.002 – 0.05)	0.55 (0.40 -0.69)	0.74 (0.57 – 0.95)	1.48 (1.18 – 1.84)
FLW advised adoption of sterilization	0.05 (0.02 – 0.10)	0.59 (0.46 – 0.71)	0.76 (0.62 – 0.90)	1.30 (1.08 – 1.57)
Use of modern method of contraception	0.09 (0.05 – 0.14)	0.24 (0.18 – 0.30)	0.91 (0.83 – 0.99)	1.02 (0.94 – 1.14)

Figure S1: Round 6 and round 9 indicator levels by block

<table>
<tr><th>Round 6</th><th>Round 9</th></tr>
<tr><td>
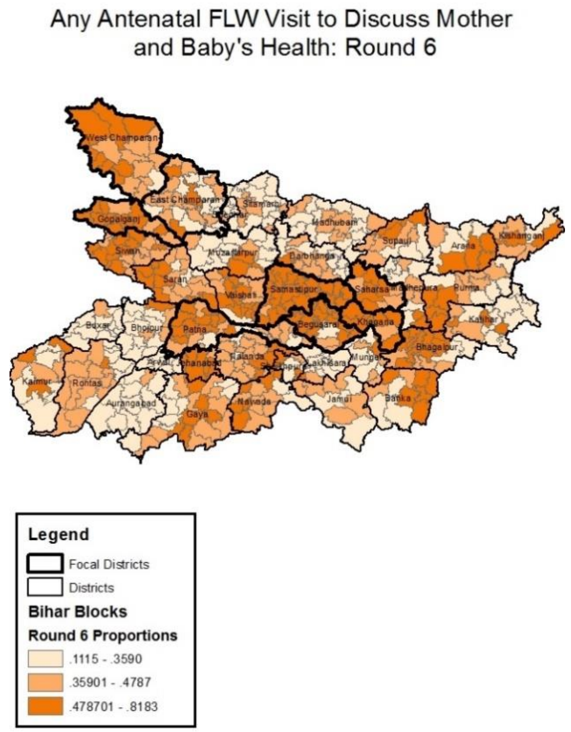

</td><td>
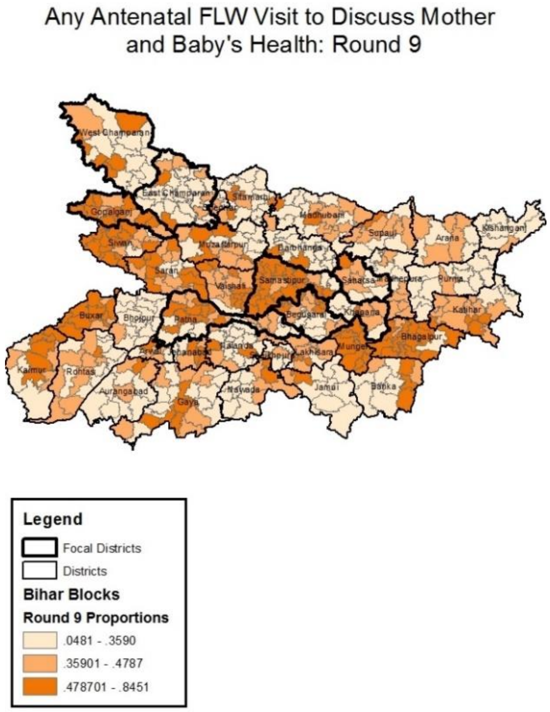

</td></tr>
<tr><td>
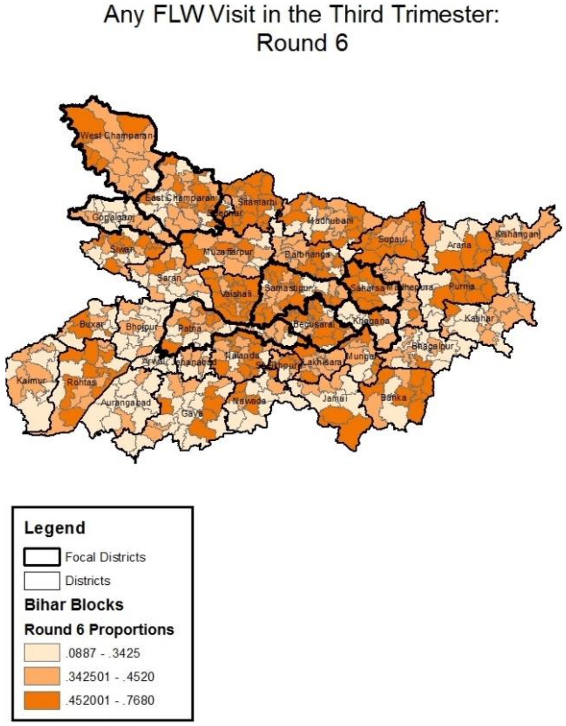

</td><td>
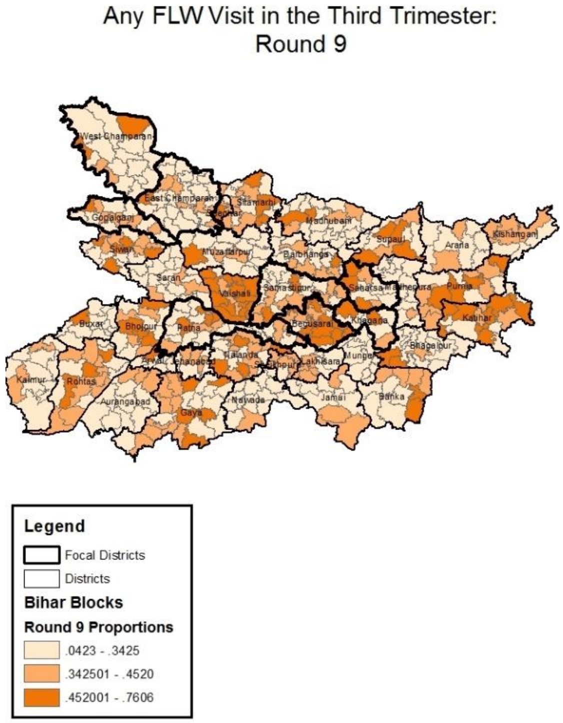

</td></tr>
</table>

<table>
<tr><th>Round 6</th><th>Round 9</th></tr>
<tr><td>
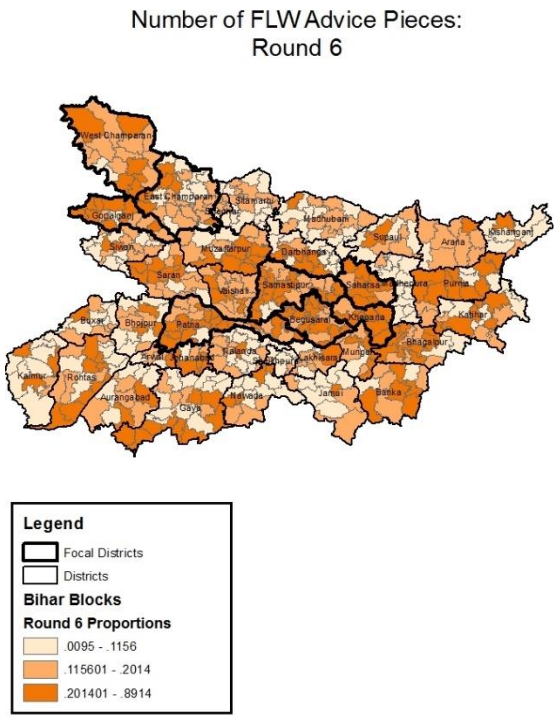

</td><td>
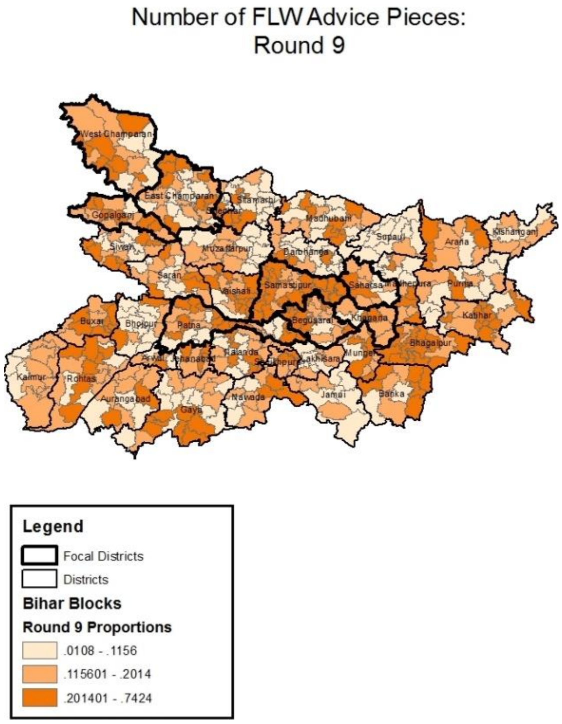

</td></tr>
<tr><td>
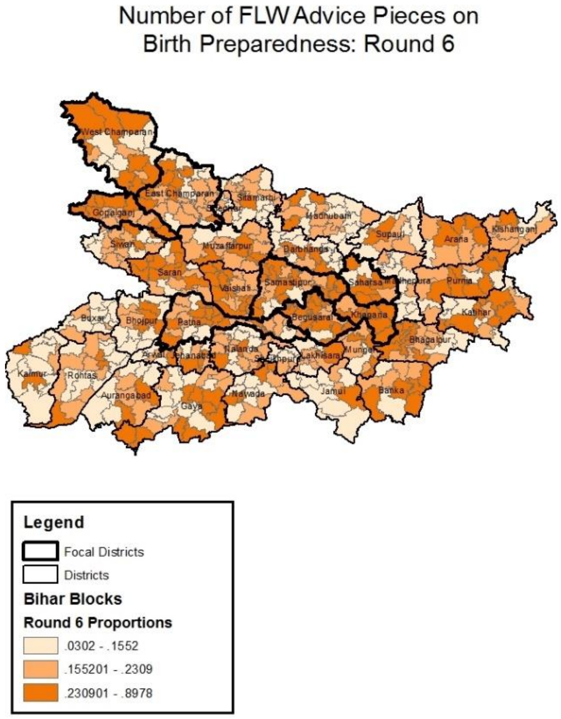

</td><td>
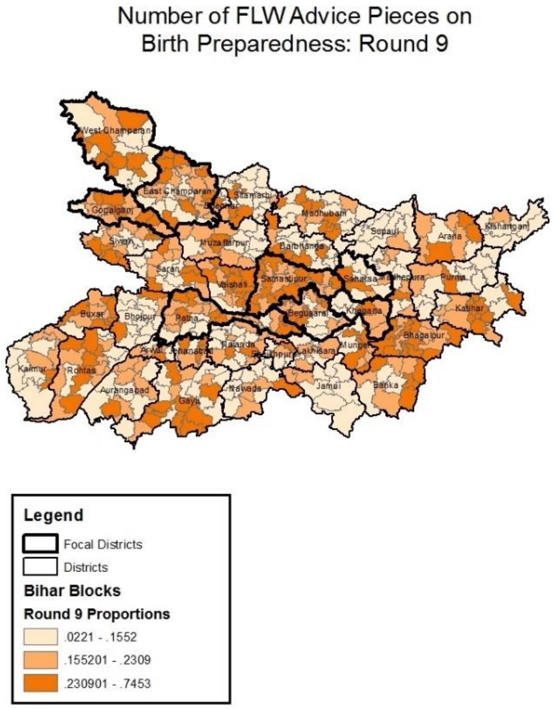

</td></tr>
</table>

Round 6	Round 9

FLW Advised on Identifying Pregnancy Danger Signs: Round 6

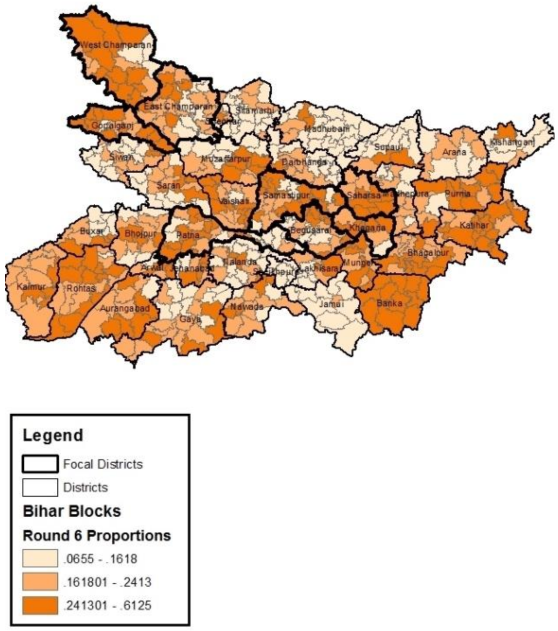

FLW Advised on Identifying Pregnancy Danger Signs: Round 9

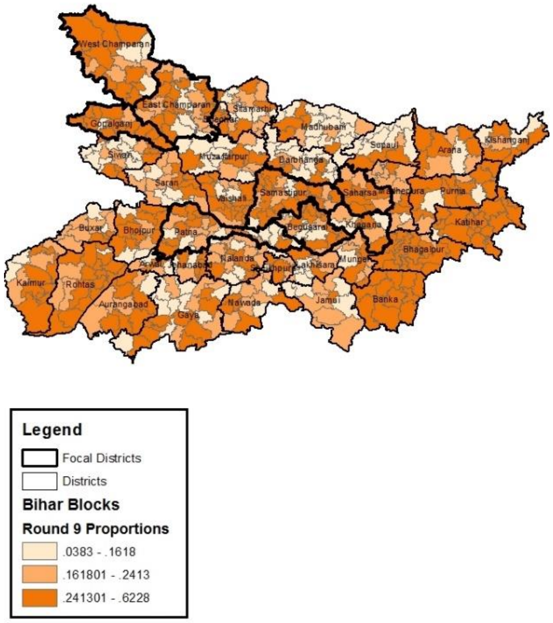

Number of Birth Preparedness Measures Taken: Round 6

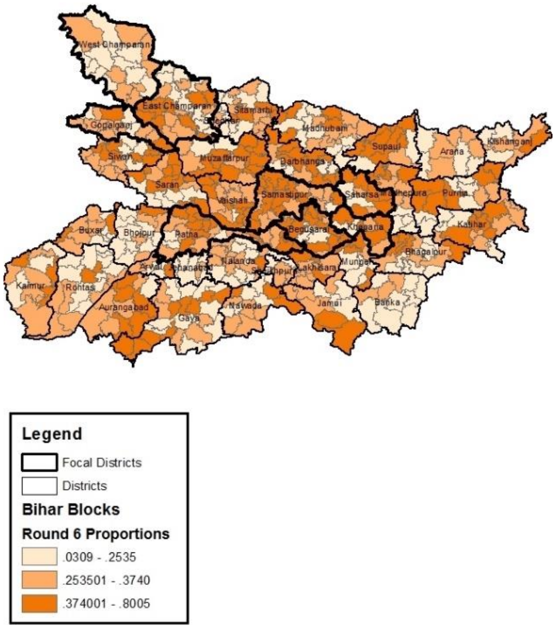

Number of Birth Preparedness Measures Taken: Round 9

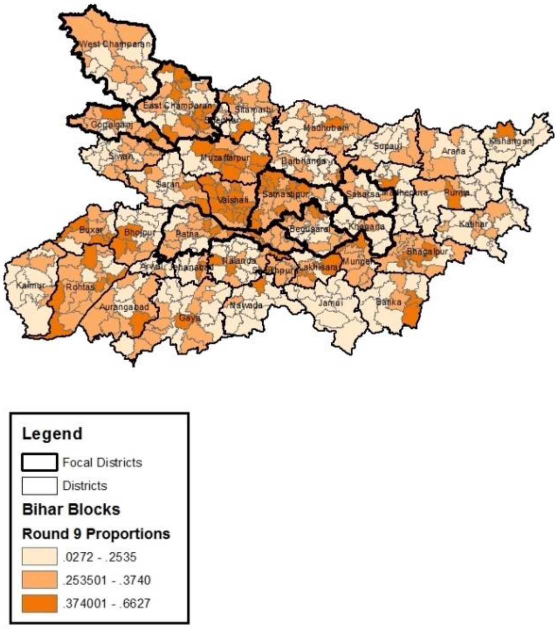

Round 6	Round 9

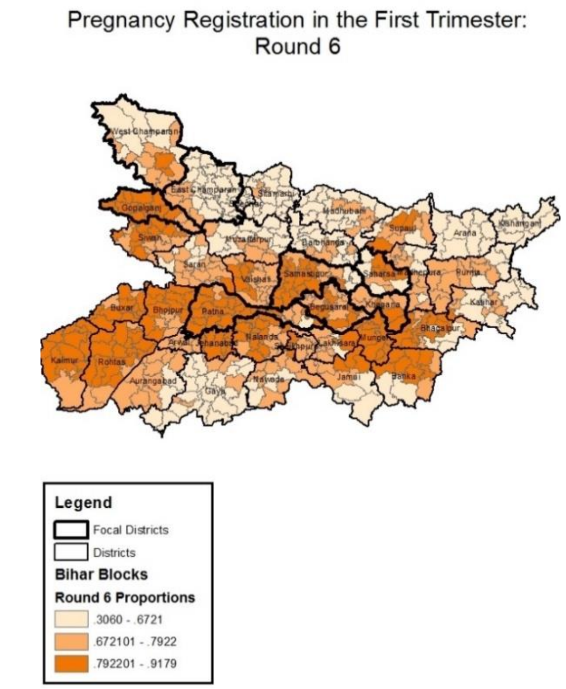

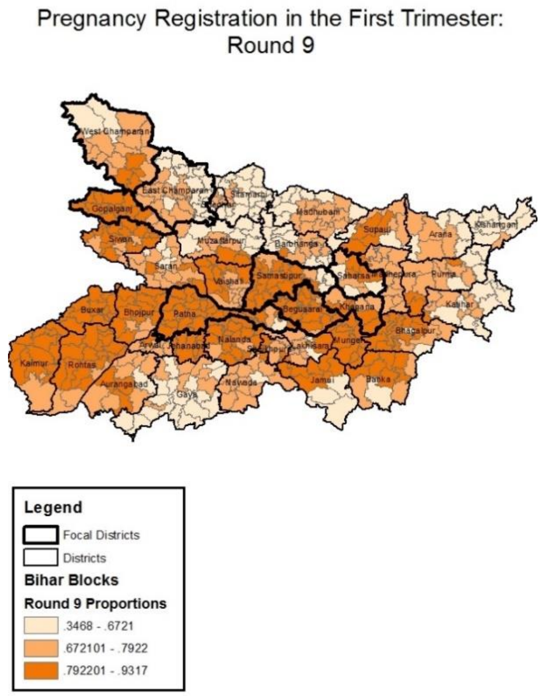

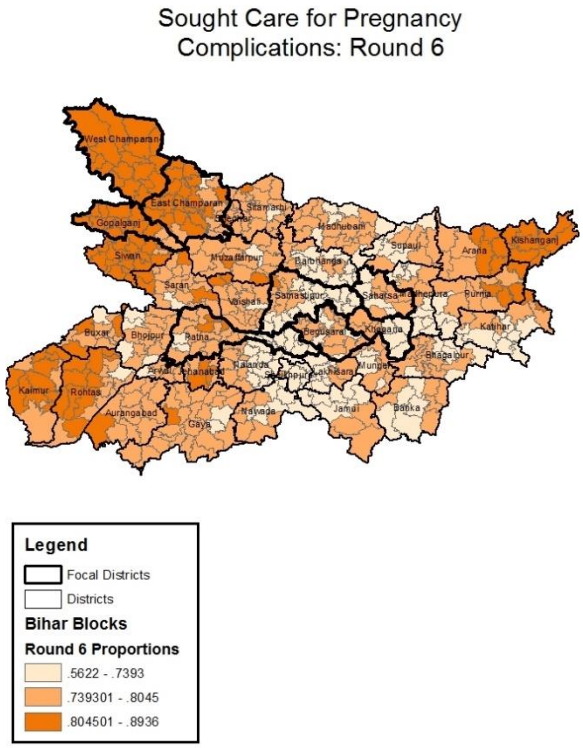

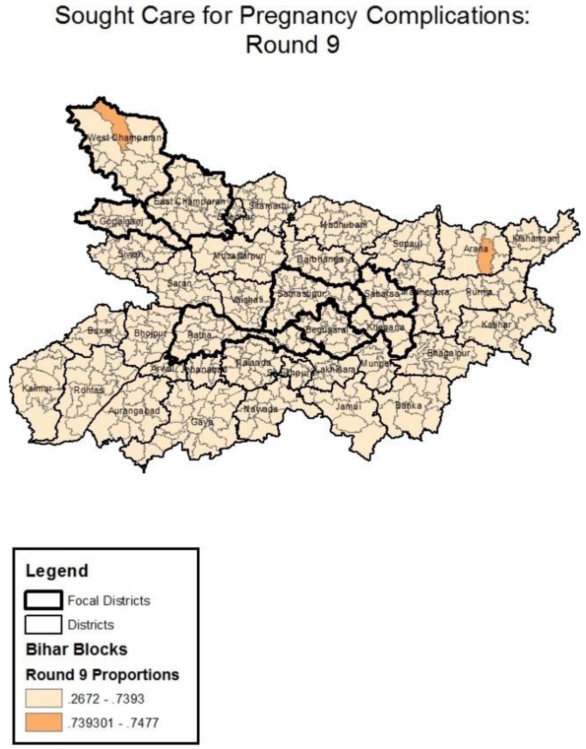

Round 6	Round 9

4+ ANC Visits: Round 9

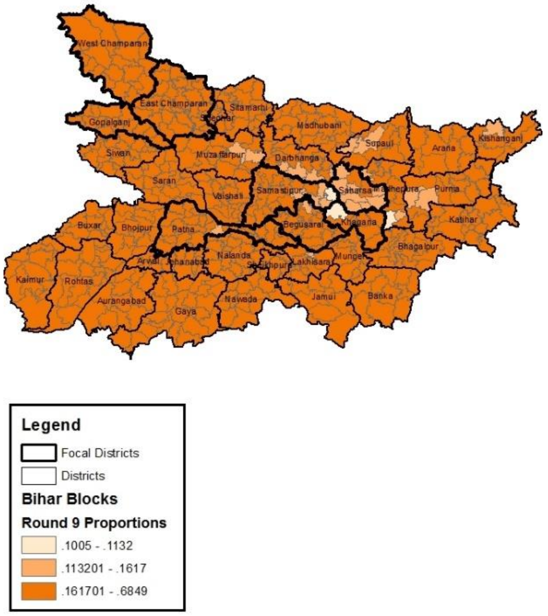

4+ ANC Visits: Round 6

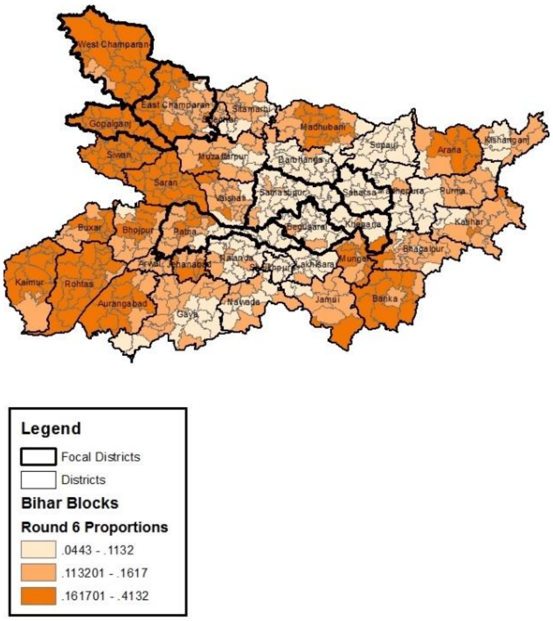

Received 90+ IFA: Round 6

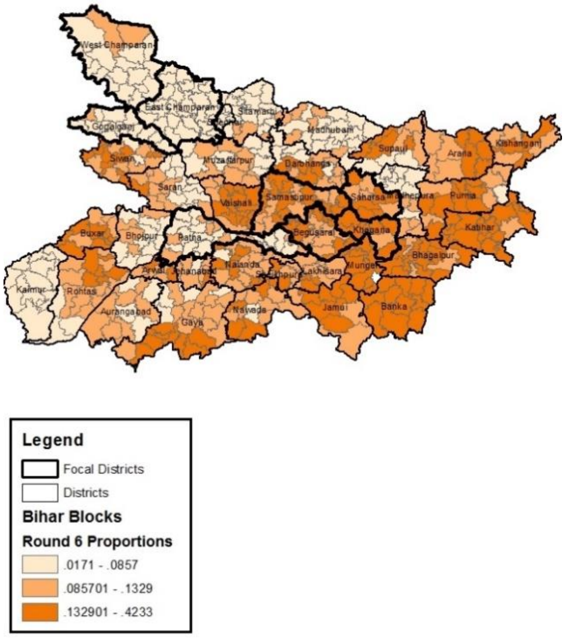

Received 90+ IFA: Round 9

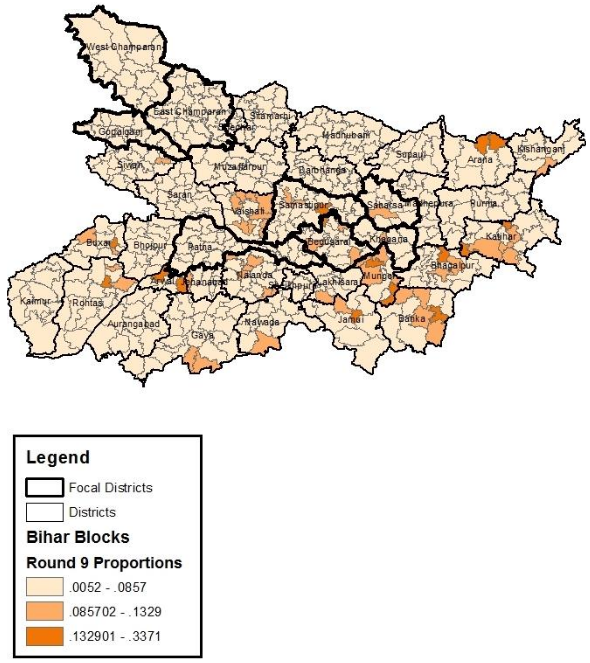

Round 6	Round 9

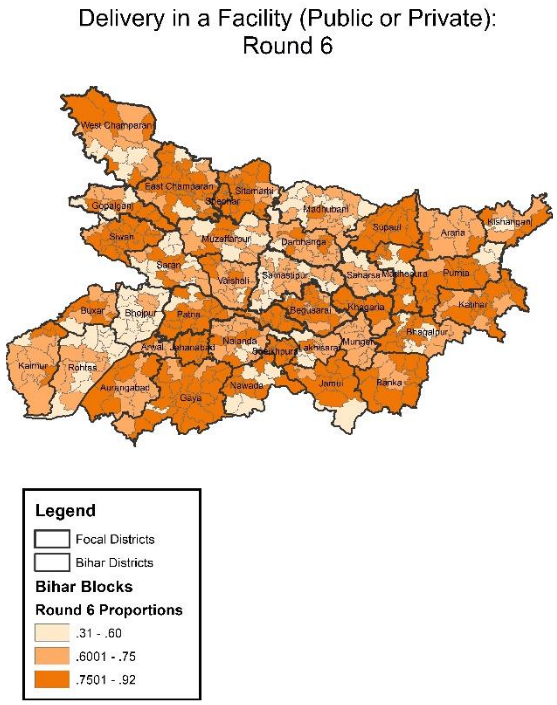

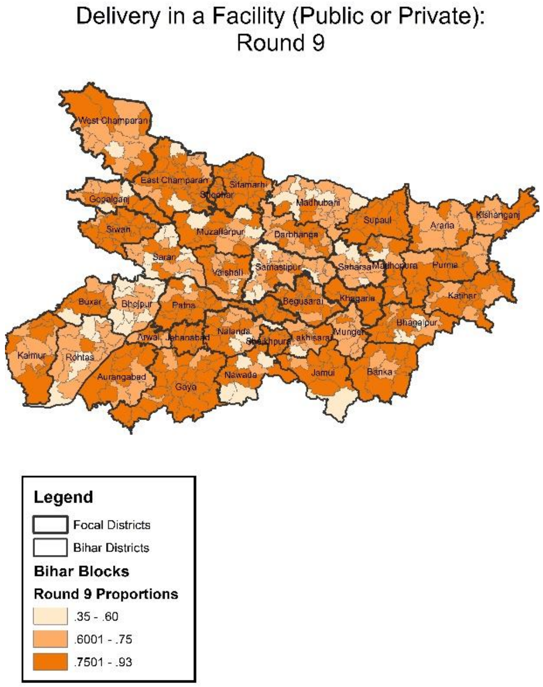

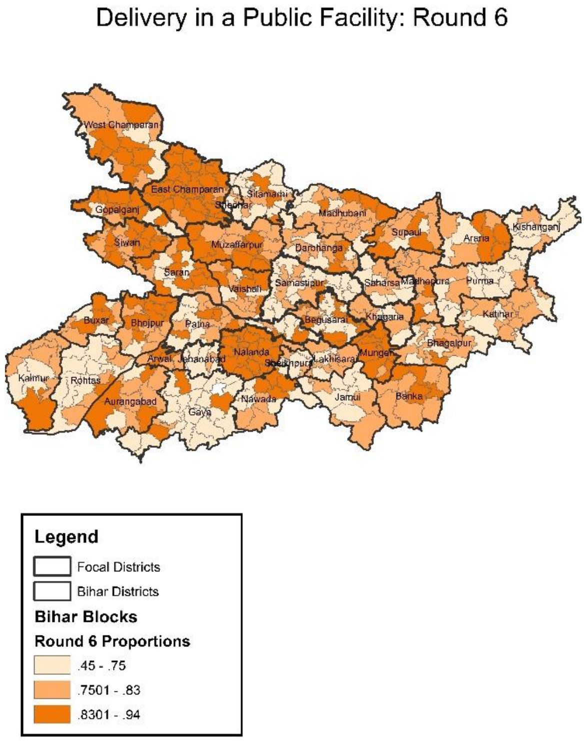

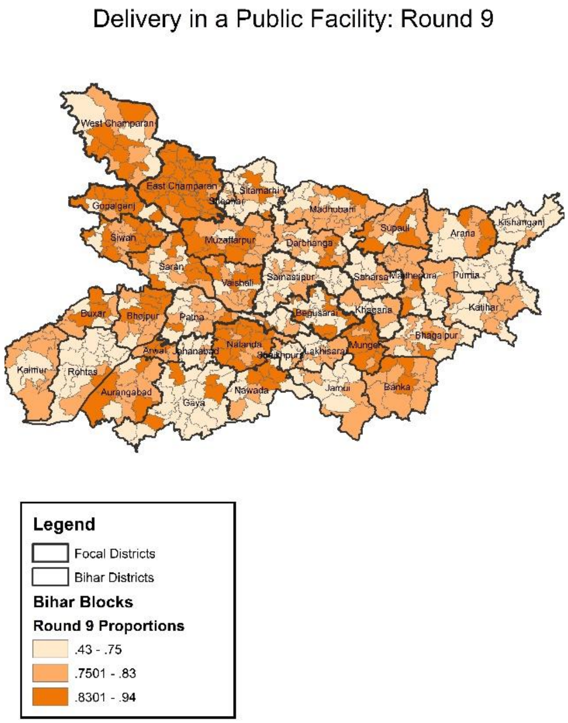

<table>
<tr><th>Round 6</th><th>Round 9</th></tr>
<tr><td>
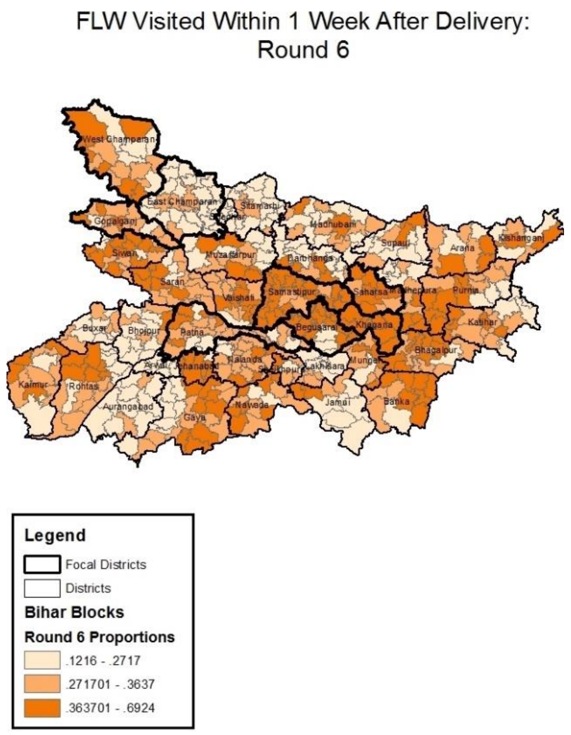

</td><td>
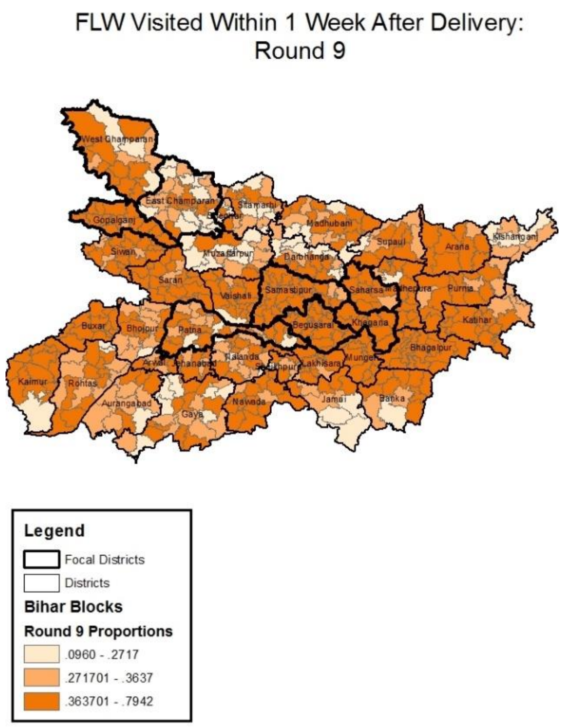

</td></tr>
<tr><td>
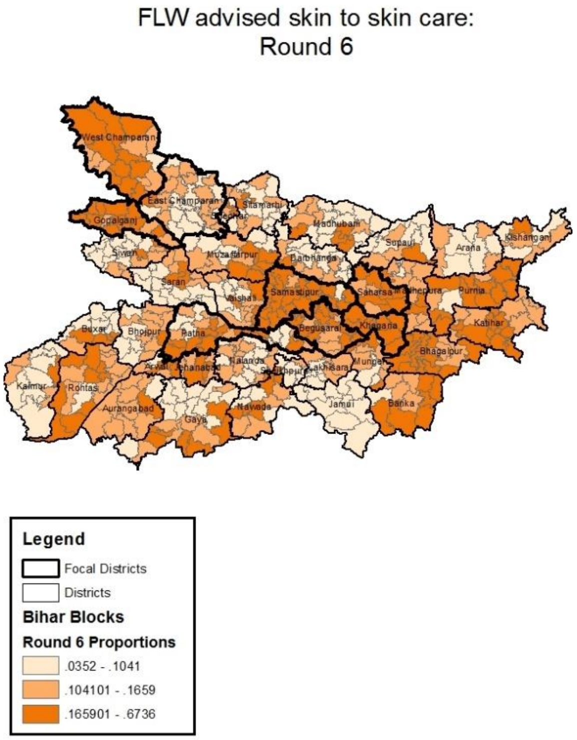

</td><td>
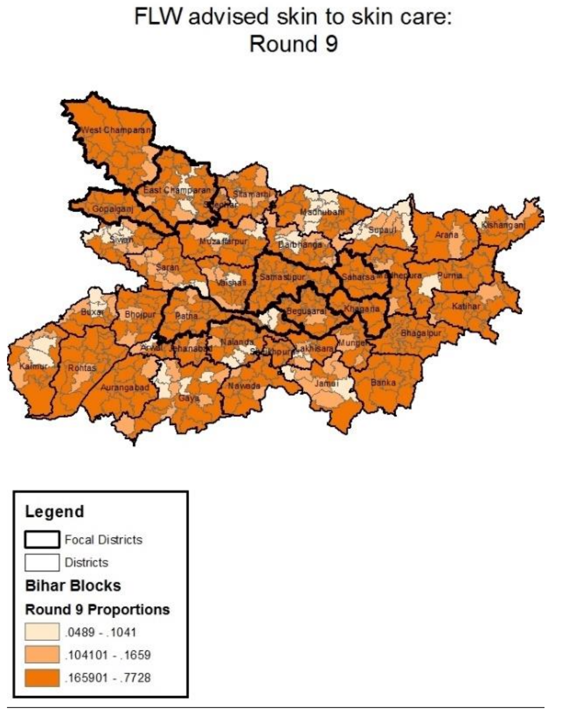

</td></tr>
</table>

<table>
<tr><th>Round 6</th><th>Round 9</th></tr>
<tr><td>
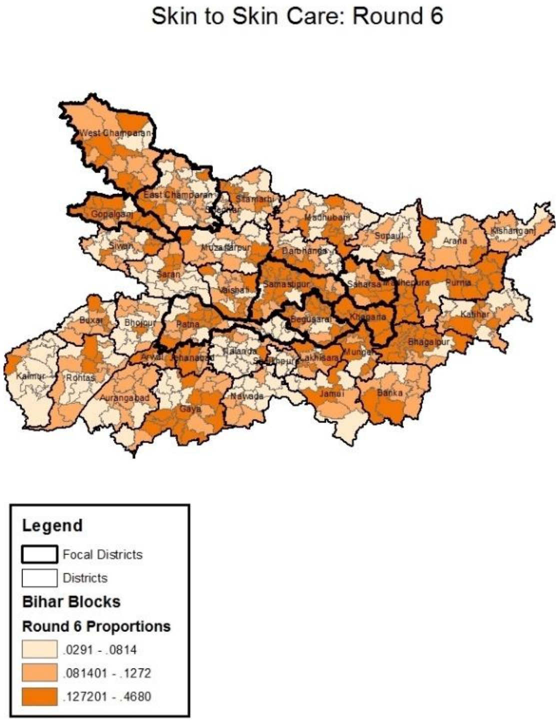

</td><td>
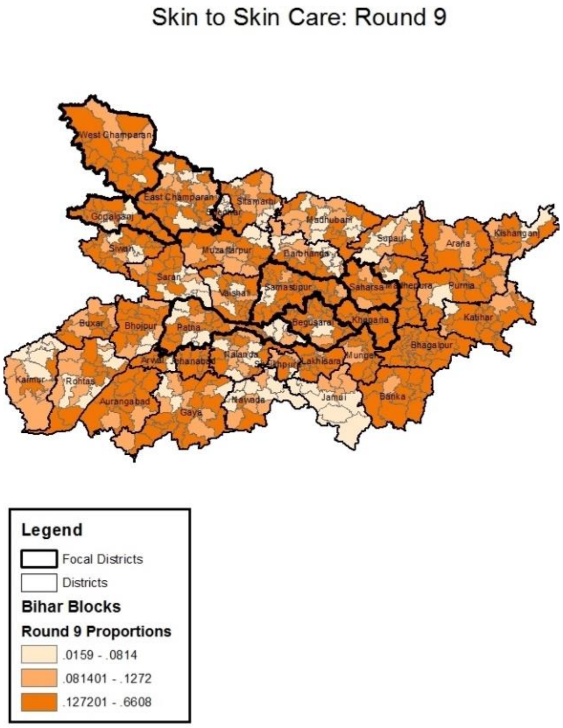

</td></tr>
<tr><td>
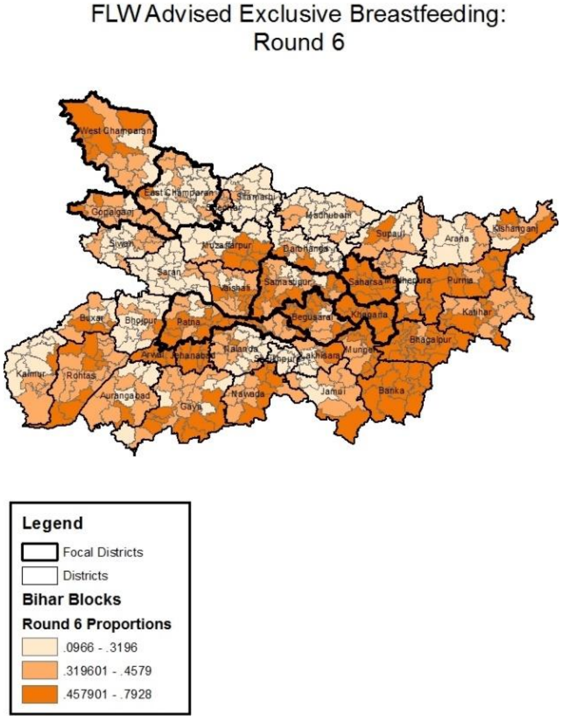

</td><td>
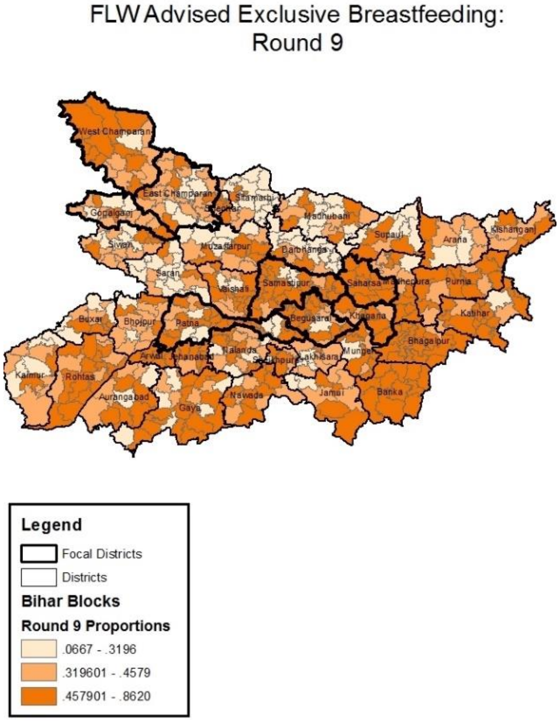

</td></tr>
</table>

<table>
<tr><th>Round 6</th><th>Round 9</th></tr>
<tr><td>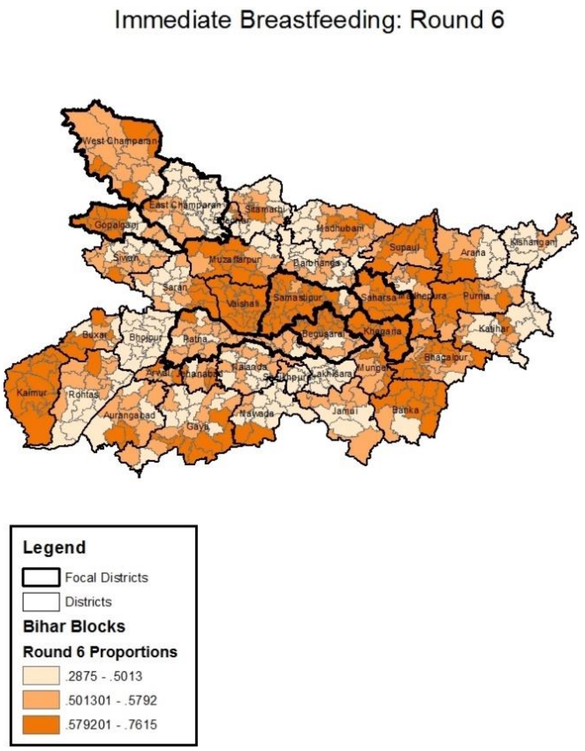
</td><td>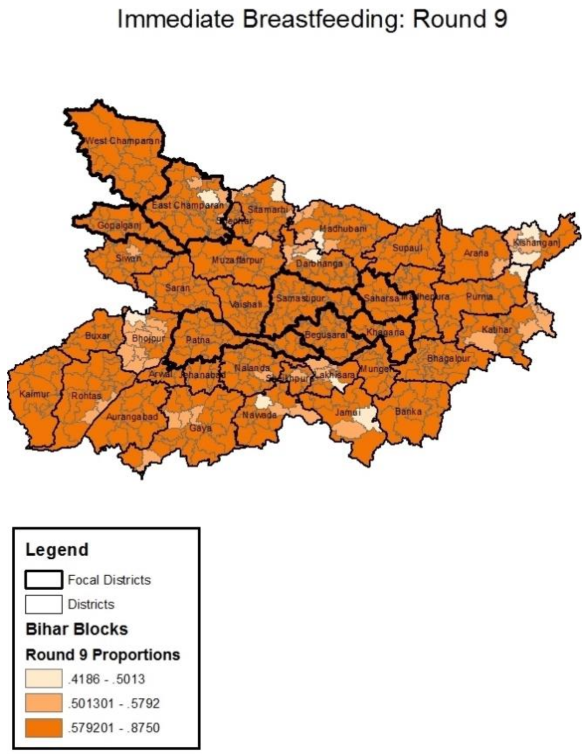
</td></tr>
<tr><td>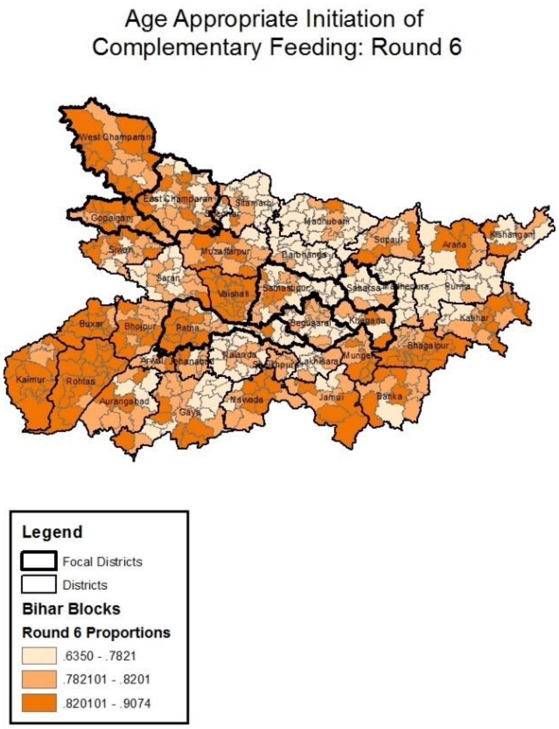
</td><td>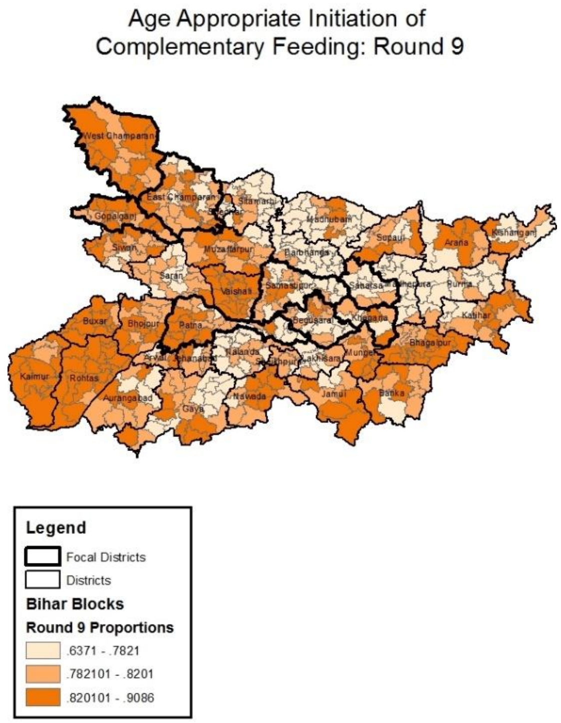
</td></tr>
</table>

Round 6	Round 9

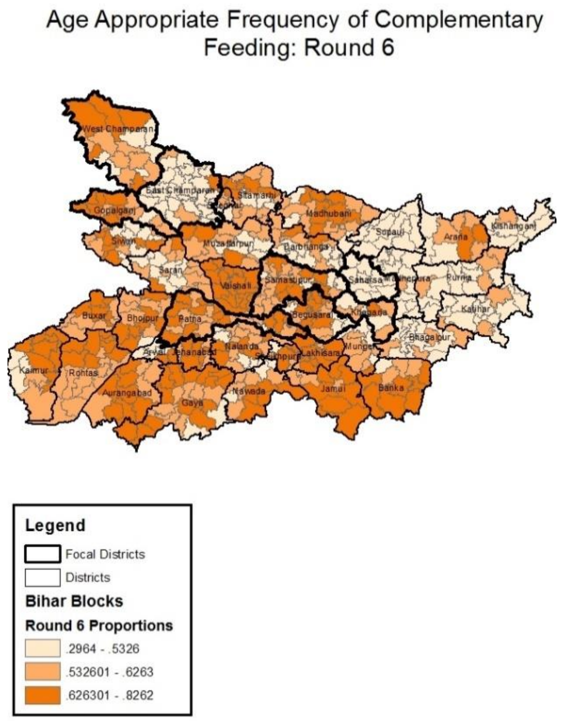

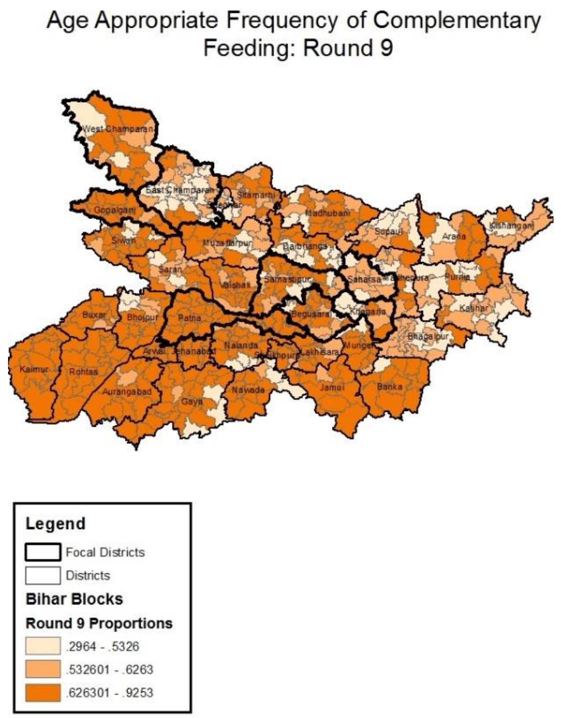

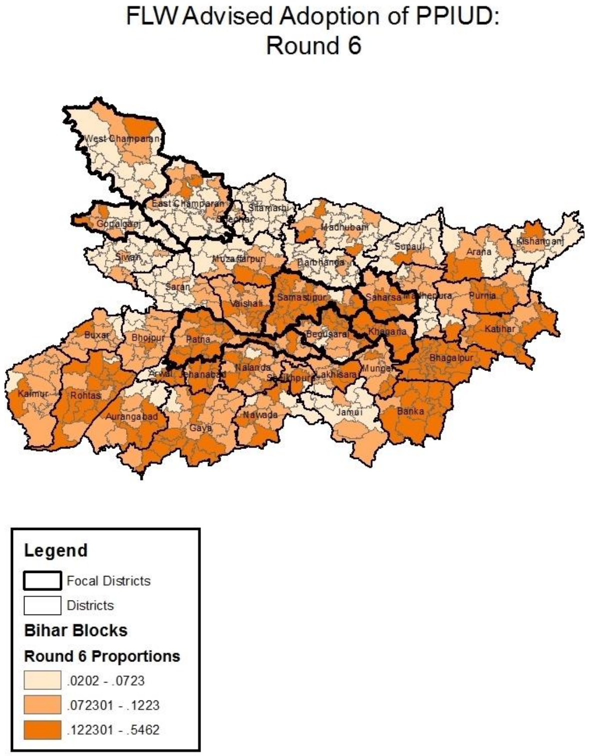

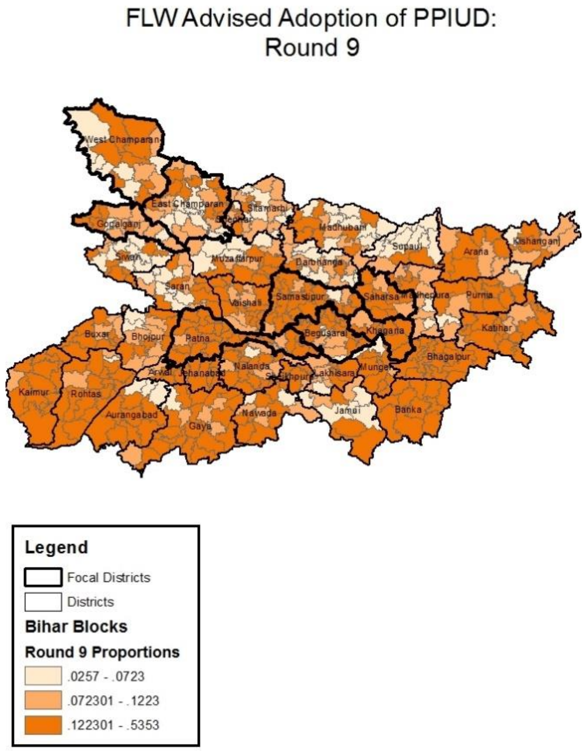

<table>
<tr><th>Round 6</th><th>Round 9</th></tr>
<tr><td>
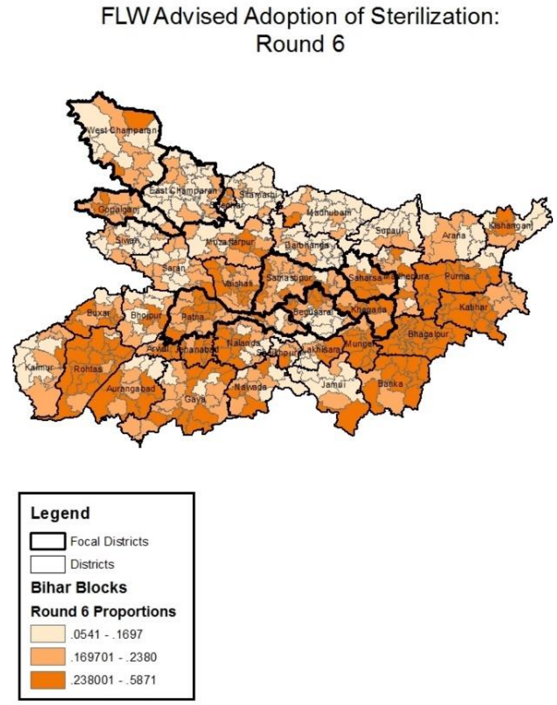

</td><td>
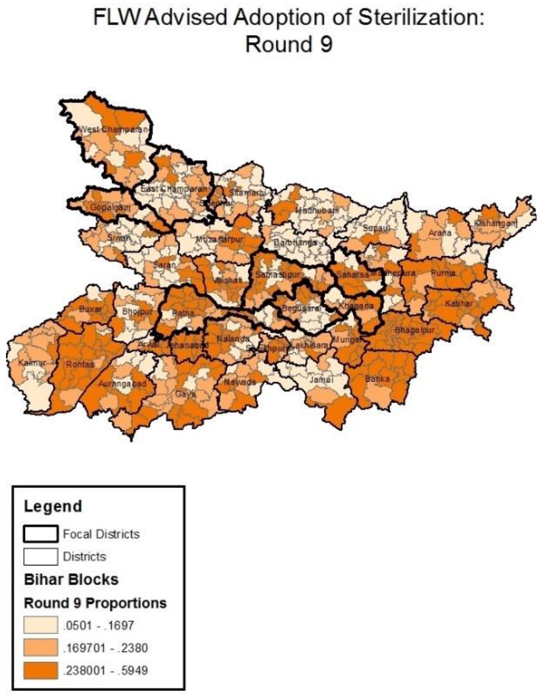

</td></tr>
<tr><td>
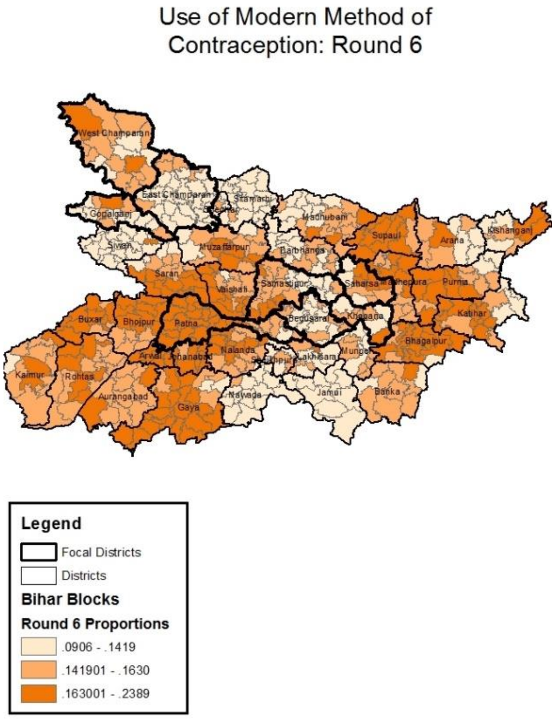

</td><td>
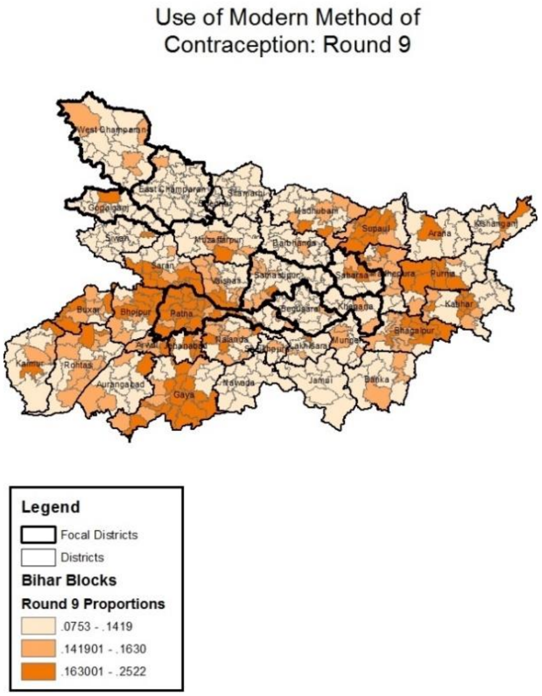

</td></tr>
</table>

Figure S2: distribution of the initial level of indicator in 2014 (round 6), CHS, Bihar

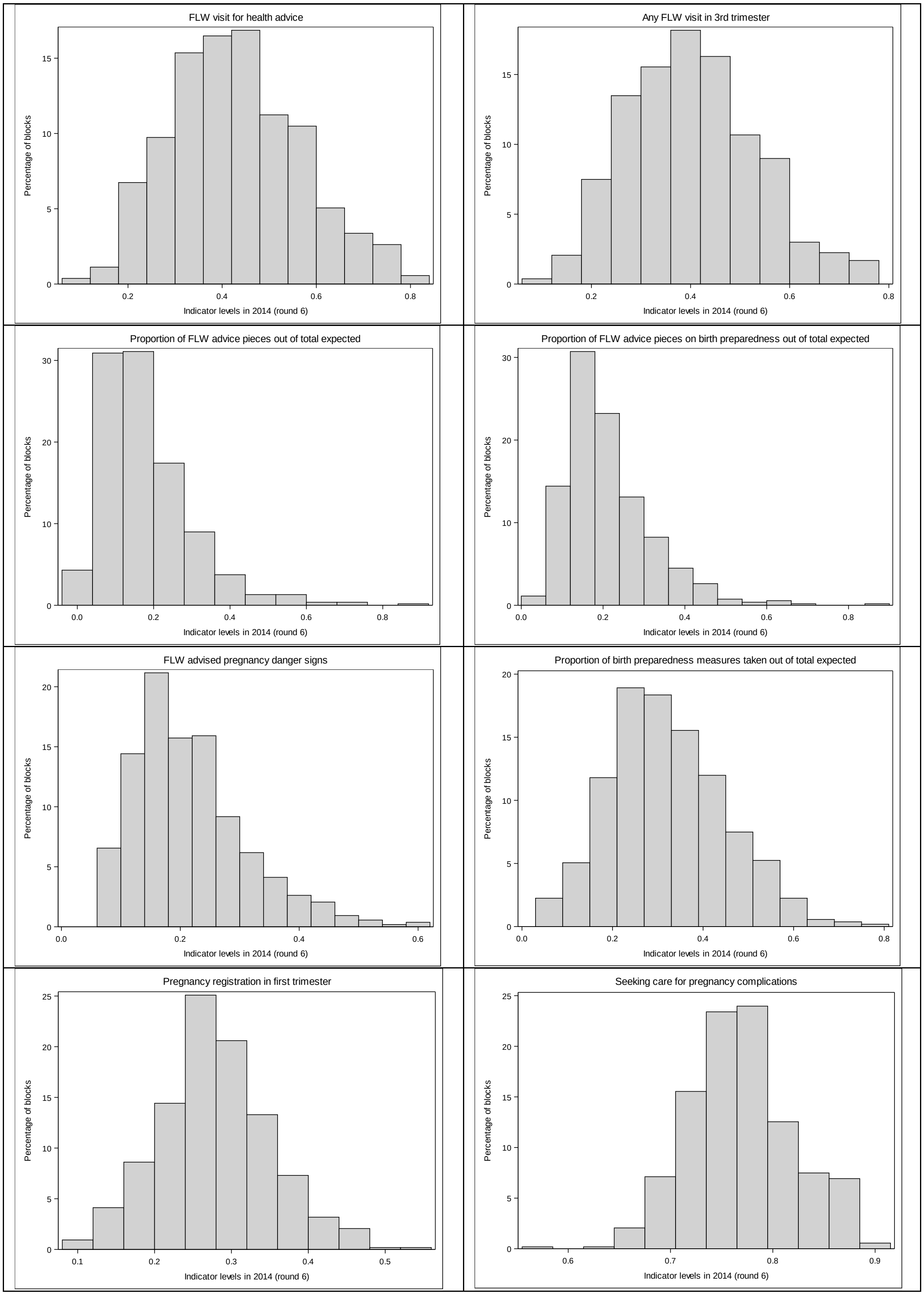

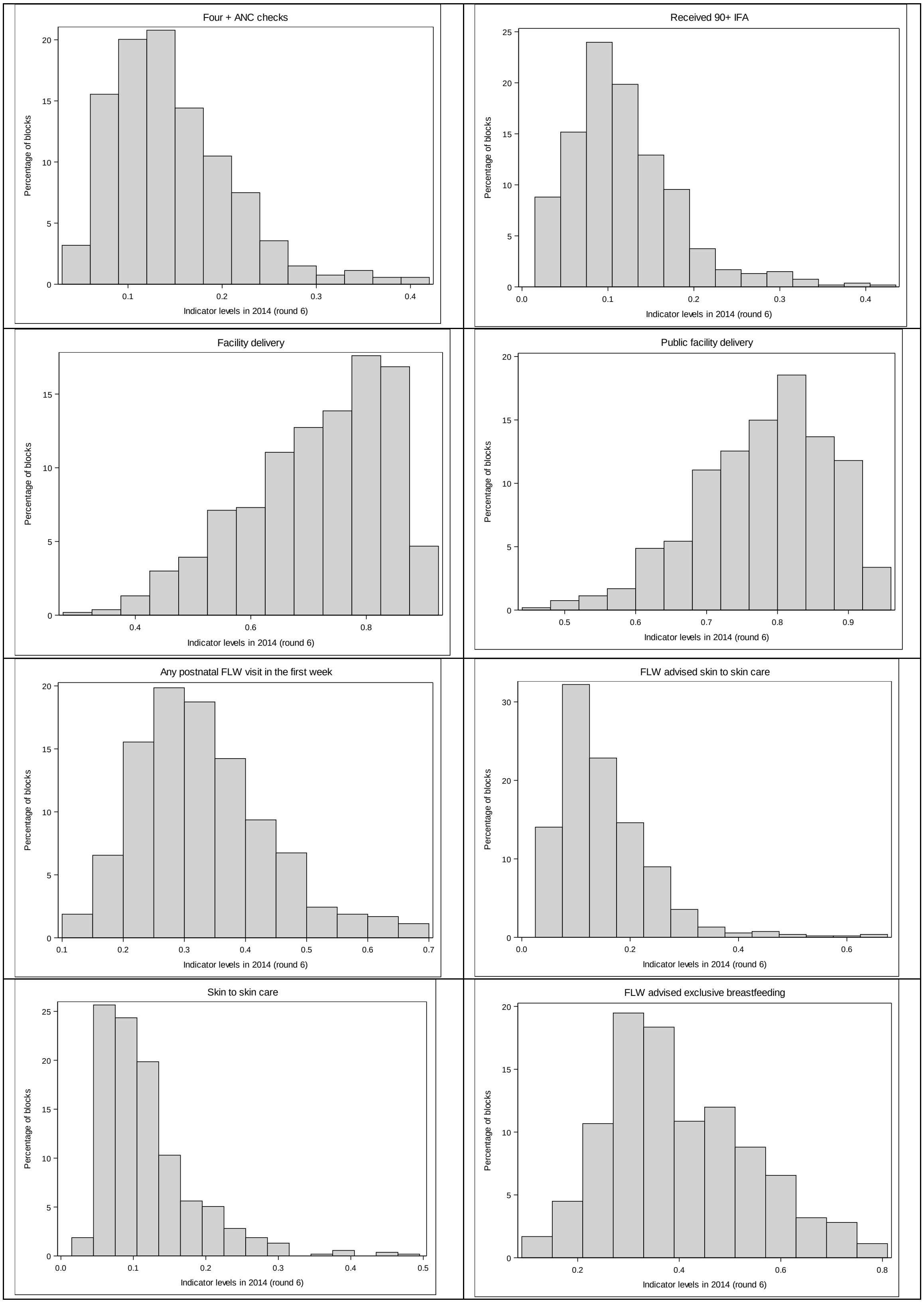

Four + ANC checks
Percentage of blocks
Indicator levels in 2014 (round 6)
Received 90+ IFA
Percentage of blocks
Indicator levels in 2014 (round 6)
Facility delivery
Percentage of blocks
Indicator levels in 2014 (round 6)
Public facility delivery
Percentage of blocks
Indicator levels in 2014 (round 6)
Any postnatal FLW visit in the first week
Percentage of blocks
Indicator levels in 2014 (round 6)
FLW advised skin to skin care
Percentage of blocks
Indicator levels in 2014 (round 6)
Skin to skin care
Percentage of blocks
Indicator levels in 2014 (round 6)
FLW advised exclusive breastfeeding
Percentage of blocks
Indicator levels in 2014 (round 6)

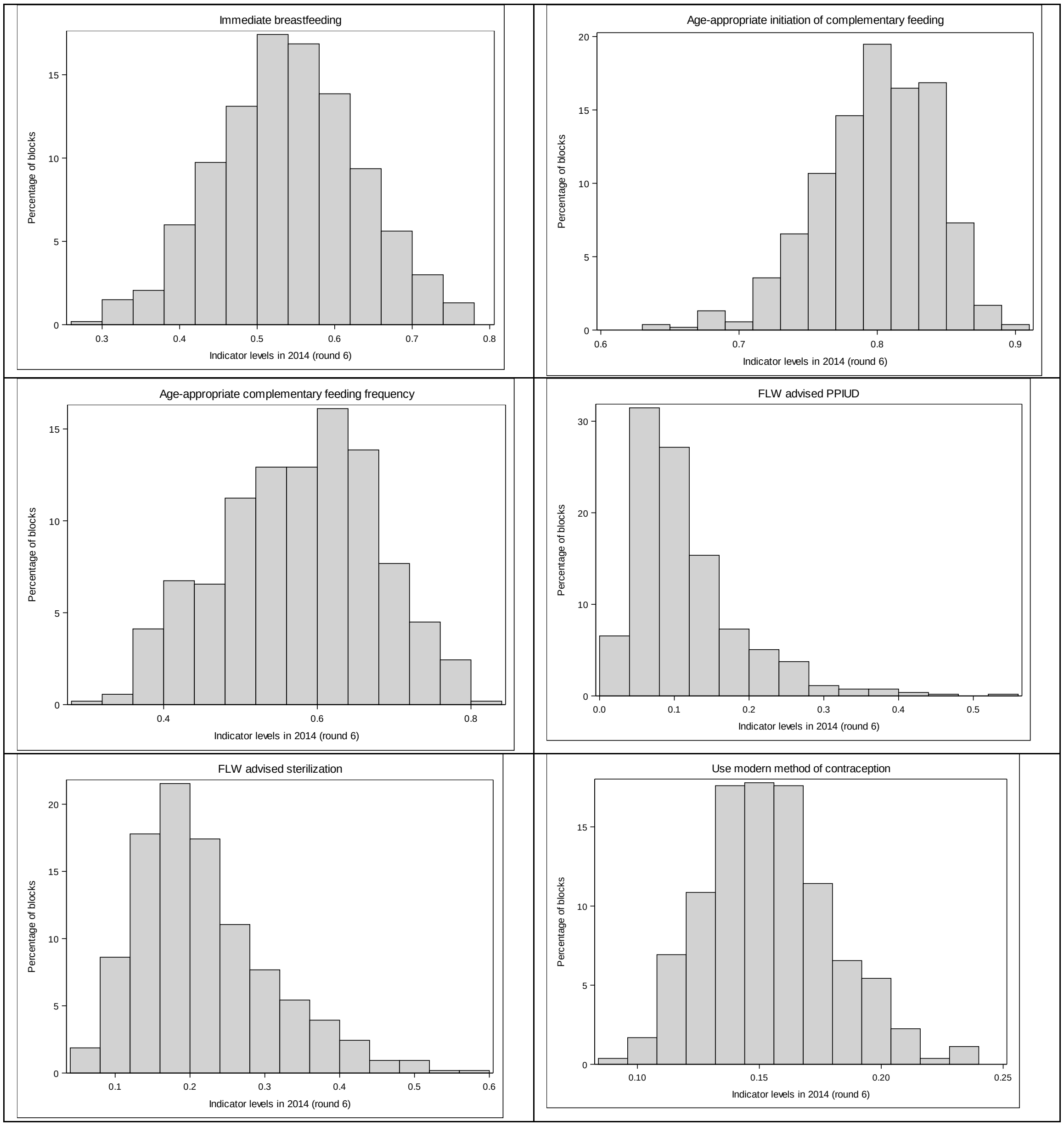
Immediate breastfeeding
Percentage of blocks
Indicator levels in 2014 (round 6)
Age-appropriate initiation of complementary feeding
Percentage of blocks
Indicator levels in 2014 (round 6)
Age-appropriate complementary feeding frequency
Percentage of blocks
Indicator levels in 2014 (round 6)
FLW advised PPIUD
Percentage of blocks
Indicator levels in 2014 (round 6)
FLW advised sterilization
Percentage of blocks
Indicator levels in 2014 (round 6)
Use modern method of contraception
Percentage of blocks
Indicator levels in 2014 (round 6)

Figure S3: number of indicators per block where the relative change per year was more than 1 or less than 1 with 97.5% or more probability

Relative change more than 1 with 97.5% probability	Relative change per year less than 1 with 97.5% probability

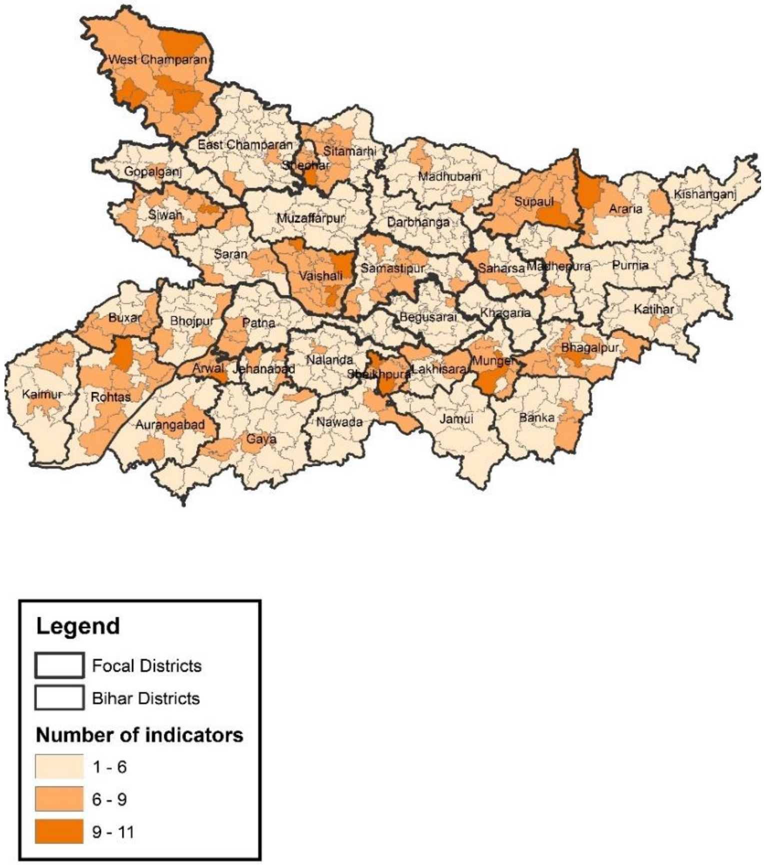

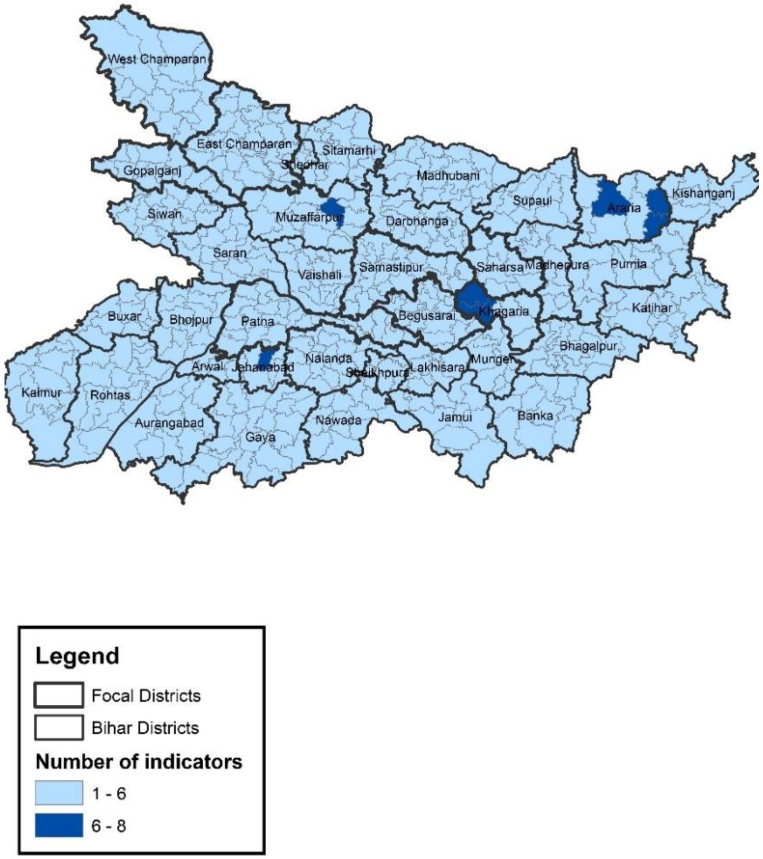

Supplement: Online Supplementary Document [file jogh-10-021004-s001.pdf]
